# Association of dietary inflammatory potential with cardiometabolic risk factors and diseases: a systematic review and dose–response meta-analysis of observational studies

**DOI:** 10.1186/s13098-020-00592-6

**Published:** 2020-10-07

**Authors:** Zahra Aslani, Omid Sadeghi, Motahar Heidari-Beni, Hoda Zahedi, Fereshteh Baygi, Nitin Shivappa, James R. Hébert, Sajjad Moradi, Gity Sotoudeh, Hamid Asayesh, Shirin Djalalinia, Mostafa Qorbani

**Affiliations:** 1 Department of Community Nutrition, School of Nutritional Sciences and Dietetics, Tehran University of Medical Sciences, Tehran, Iran; 2 Students’ Scientific Research Center, Tehran University of Medical Sciences, Tehran, Iran; 3 Child Growth and Development Research Center, Research Institute for Primordial Prevention of Non-Communicable Disease, Isfahan University of Medical Sciences, Isfahan, Iran; 4 Hematopoietic Stem Cell Research Center, Shahid Beheshti University of Medical Sciences, Tehran, Iran; 5 Center of Maritime Health and Society, Department of Public Health, University of Southern Denmark, Esbjerg, Denmark; 6 Cancer Prevention and Control Program, University of South Carolina, Columbia, SC 29208 USA; 7 Department of Epidemiology and Biostatistics, Arnold School of Public Health, University of South Carolina, Columbia, SC 29208 USA; 8 Connecting Health Innovations LLC, Columbia, SC 29201 USA; 9 Halal Research Center of IRI, FDA, Tehran, Iran; 10 Department of Clinical Nutrition, School of Nutrition and Food Science, Isfahan University of Medical Sciences, Isfahan, Iran; 11 Department of Medical Emergencies, Qom University of Medical Sciences, Qom, Iran; 12 Development of Research & Technology Center, Ministry of Health and Medical Education, Tehran, Iran; 13 Non-Communicable Diseases Research Center, Endocrinology and Metabolism Population Sciences Institute, Tehran University of Medical Sciences, Tehran, Iran; 14 Non-Communicable Diseases Research Center, Alborz University of Medical Sciences, Karaj, Iran; 15 Chronic Diseases Research Center, Endocrinology and Metabolism Population Sciences Institute, Tehran University of Medical Sciences, Tehran, Iran

**Keywords:** Diet, Inflammation, Cardiovascular diseases, Dietary inflammatory index

## Abstract

**Context:**

The association of dietary inflammatory index (DII®), as an index of inflammatory quality of diet, with cardiometabolic diseases (CMDs) and risk factors (CMRFs) has been inconsistent in previous studies.

**Objective:**

The current systematic review and dose–response meta-analysis was performed to investigate the association of the DII score with CMDs and CMRFs.

**Data Sources:**

All published observational studies (cohort, case–control and cross-sectional) using PubMed/Medline, Scopus, ISI Web of Science, and Google Scholar databases were retrieved from inception through November 2019.

**Data extraction:**

Two reviewers independently extracted the data from included studies.

**Data analysis:**

Pooled hazard ratio (HR) or odds ratio (OR) were calculated by using a random-effects model.

**Results:**

Ten prospective cohort studies (total n = 291,968) with 31,069 CMDs-specific mortality, six prospective cohort studies (total n = 43,340) with 1311 CMDs-specific morbidity, two case–control studies with 2140 cases and 6246 controls and one cross-sectional study (total n = 15,613) with 1734 CMDs-specific morbidity were identified for CMDs. Meta-analyses of published observational studies demonstrated that the highest DII score category versus the lowest DII score category was associated with 29% increased risk of CMDs mortality (HR = 1.29; 95% confidence interval (CI) 1.18, 1.41). Moreover, there was a significant association between the DII score and risk of CMDs in cohort studies (HR = 1.35; 95% CI 1.13, 1.61) and non-cohort study (HR = 1.36; 95% CI 1.18, 1.57). We found a significant association between the DII score and metabolic syndrome (MetS) (OR: 1.13; 95% CI 1.03, 1.25), hyperglycemia and hypertension. None-linear dose response meta-analysis showed that there was a significant association between the DII score and risk of CMDs mortality (P_nonlinearity_ < 0.001). Moreover, evidence of none-linear association between the DII score and risk of CMDs was not observed (p-value = 0.1).

**Conclusions:**

Adherence to pro-inflammatory diet was associated with increased risk of CMDs, mortality and MetS.

## Background

Chronic inflammation happens through frequent stress factors such as poor diet and obesity [1] and it is recognized with high levels of serum inflammatory biomarkers including high sensitivity C-reactive protein (hs-CRP), interleukin (IL)-6, and tumor necrosis factor-α (TNF-α). This situation is associated with chronic outcomes including cardiovascular diseases (CVDs) [2], type 2 diabetes mellitus [3], cancer [4], obesity [5], and metabolic syndrome (MetS) and its components [6]. The association of diet with inflammation and CVDs is well demonstrated in previous studies. Adherence to Mediterranean diet, which is characterized by high intake of fruits and vegetables, whole grains, legumes, nuts, fish, and olive oil, decreases chronic inflammation and is associated with lower risk of CVDs [7–11], whereas intake of foods with high amount of sugar, refined grains, red and processed meat, foods with high saturated and trans fatty acids, and sodium (Western diet) is associated with higher levels of chronic inflammation and intermediate markers of CVDs [12].

The dietary inflammatory index (DII) is a novel and validated tool designed in 2009 [13] and updated in 2014 to estimate the inflammatory potential of an individual’s diet [14]. According to this index, the food items, macronutrients, and micronutrients (45 food parameters) based on their effect on inflammatory biomarkers (IL-1β, IL-4, IL-6, IL-10, TNF-α, and CRP) were classified into pro-inflammatory, anti-inflammatory, and inflammatory neutral [14].

Multiple studies have assessed the association of the DII score with different chronic diseases [15–18] and their risk factors [19–23]; however, findings are conflicting. Various studies showed the association between the DII score and cardiometabolic risk factors (CMRFs) such as MetS [23], hypertension (HTN) [17, 24], and serum glucose levels [20], while other studies did not show this association [25–28]. Several observational reports have demonstrated the obvious association of the DII score with cardiometabolic diseases (CMDs)-specific morbidity and mortality [15, 19, 29, 30], whereas other studies failed to find any association [31, 32].

Given the inconsistent findings, this meta-analysis was conducted to summarize the association of DII with CMRFs and CMDs in observational studies.

Although recently some systematic reviews and meta-analyses have addressed the association between the DII score and CVDs morbidity and mortality [33–35] and MetS [34], none of them has evaluated the association of DII score with cardio-metabolic risk factors (e.g. lipid profile, glycemic indices, and anthropometric measures). Moreover, there is no comprehensive systematic review of assessing the association of both continuous and categorical DII score variables with CMRFs (e.g. lipid profile, glycemic indices, anthropometric measures, blood pressure (BP), and metabolic syndrome) and CMDs-specific morbidity and mortality. Therefore, the aim of this systematic review and meta-analysis study was to assess the association of both continuous and categorical DII score variables with risk of CMRFs and risk of CMDs and mortality.

## Methods

We followed the Preferred Reporting Items for Systematic Reviews and Meta-Analyses (PRISMA) statement for reporting in the current systematic review and mate-analysis study (Additional file 1: Appendix S1).

### Search strategy

Published reports with the aim of studying the association of DII score with CMRFs (e.g. glycemic indices, lipid profiles, anthropometric measures, MetS and its components) and CMDs (like MI, IHD, stroke, congestive heart failure, and coronary heart disease (CHD) according to the International Classification of Diseases (ICD)-9-390-465) were included through comprehensive searches on PubMed and the NLM Gateway (for MEDLINE), Scopus, and Institute of Scientific Information (ISI) electronic databases up to February 2020. The appropriate medical subject headings, Entry Terms, and Emtree options were applied to carry out the most sensitive search operations. The search strategy is presented in Additional file 2: Appendix S2. A manual search was performed on Google Scholar database and the references listed in relevant reviews.

### Inclusion criteria

Two reviewers (ZA and HA) independently reviewed and screened the appropriate published papers based on title, abstract, and full text. The third reviewer (MQ) resolved any discrepancy in choosing eligible records. All observational studies (cross-sectional, case–control, and cohort) on human subjects without restriction of age group, gender, year of publication, and language examining the association between the DII score with CMRFs (e.g. glycemic indices, lipid profiles, anthropometric measures, MetS and its components) and CMDs were included in the current study.

### Exclusion criteria

The papers with the following conditions were excluded: (1) studies that considered the DII as a dependent variable, (2) letters, abstracts and reviews, and (3) duplicated publications. For multiple publications of the same population, only the article with the largest sample size was included.

The participants, intervention, comparators, outcomes, study design criteria are listed in Table 1.

**Table 1:** Participants, intervention, comparators, outcomes, study design (PICOS) criteria for inclusion of studies

Population	All population
Intervention	The DII score
Comparison	The higher DII score vs. the lower DII score
Outcome	Risk of cardiometabolic diseases and mortality
Study design	Observational studies

### Data extraction

Two investigators (ZA and SD) independently extracted the following information from each qualified study: first author, year of publication, study design, country, age range/mean age, gender, sample size, diet assessment tool, the number of subjects with abnormal CMRFs/the number of subjects with CMDs, follow-up duration, exposure variable (DII/E-DII), and the number of food items used to calculate it, the type and definition of outcome, outcome assessment method, the type of DII score variable (categorical/continuous), and effect size, study quality, and confounders. Any disagreements were removed by the third author (MQ). Studies which reported correlation or beta coefficient, were included in the systematic review and they were not entered the meta-analyses.

### Quality assessment

The quality assessment of included studies was performed by two independent reviewer using Newcastle–Ottawa Scale (NOS) [36]. This scale consists of three portions of the selection, comparability and outcomes/exposures, and the studies earned maximum nine points. In the present study, the reports with seven or more stars were assumed to have high quality. Any discrepancy between reviewers was resolved by the third reviewer (MQ).

### Statistical analysis

All observational studies with any reported effect size (odds ratio (OR), hazard ratio (HR), correlation, or Beta coefficient) were included in qualitative synthesis. Meta-analysis was performed only for studies which reported OR and HR.

In meta-analysis, we examined association of all types of DII [continuous (per one-unit increment), categorical (highest/lowest level) and dose–response association] with CMRFs and CMDs. Meta-analyses were performed separately for CMRFs morbidity, CMDs morbidity, and CMDs related mortality.

We performed random/fixed effects meta-analysis using maximally adjusted OR/HR with 95% confidence interval (CI). Heterogeneity among studies was assessed by *I*^*2*^ [37–39]. There was between-study heterogeneity if *I*^*2*^ > 50% and p < 0.1 for the result of Q test. If the results showed the heterogeneity, a random-effects model (the DerSimonian–Laird estimator) was applied to assess the pooled OR/HR. The results of the meta-analyses were schematically presented by forest plots.

Dose–response meta-analysis was performed using a method suggested by Greenland and Orsini [40] to assess the dose–response association between DII score and CMDs related morbidity and mortality. The natural logs of the HRs and their CIs across categories of the DII score were used to compute study-specific slopes (linear trends). In this method, the distribution of cases and the HRs with the variance estimates for ≥ 3 quantitative categories of exposure were required. We considered the median or mean values of the DII scores in each category to the corresponding HR for each study. For studies that reported the scores as ranges, the midpoint was estimated in each category by calculating the mean of the lower and upper bound. When the highest and lowest categories were open-ended, the length of these open-ended intervals was assumed to be the same as that of the adjacent intervals. Restricted cubic splines (three knots at fixed percentiles of 10%, 50%, and 90% of the distribution [41]) was used to examine potential nonlinear dose–response associations of the DII score with risk of CMDs and mortality.

Publication bias was examined using Egger test and funnel plots. Subgroup analysis according to the type of study design was used to examine the association between the DII score with risk of CMDs and mortality. Sensitivity analysis was performed to assess the effect of removing any of the studies or group of studies on CMDs and CMRFs. All statistical analyses were performed using Stata software version 12 (Stata Corp, College Station, Texas, USA) and p-value < 0.05 was considered statistically significant.

## Results

### Search results and study selection

**Fig. 1:**
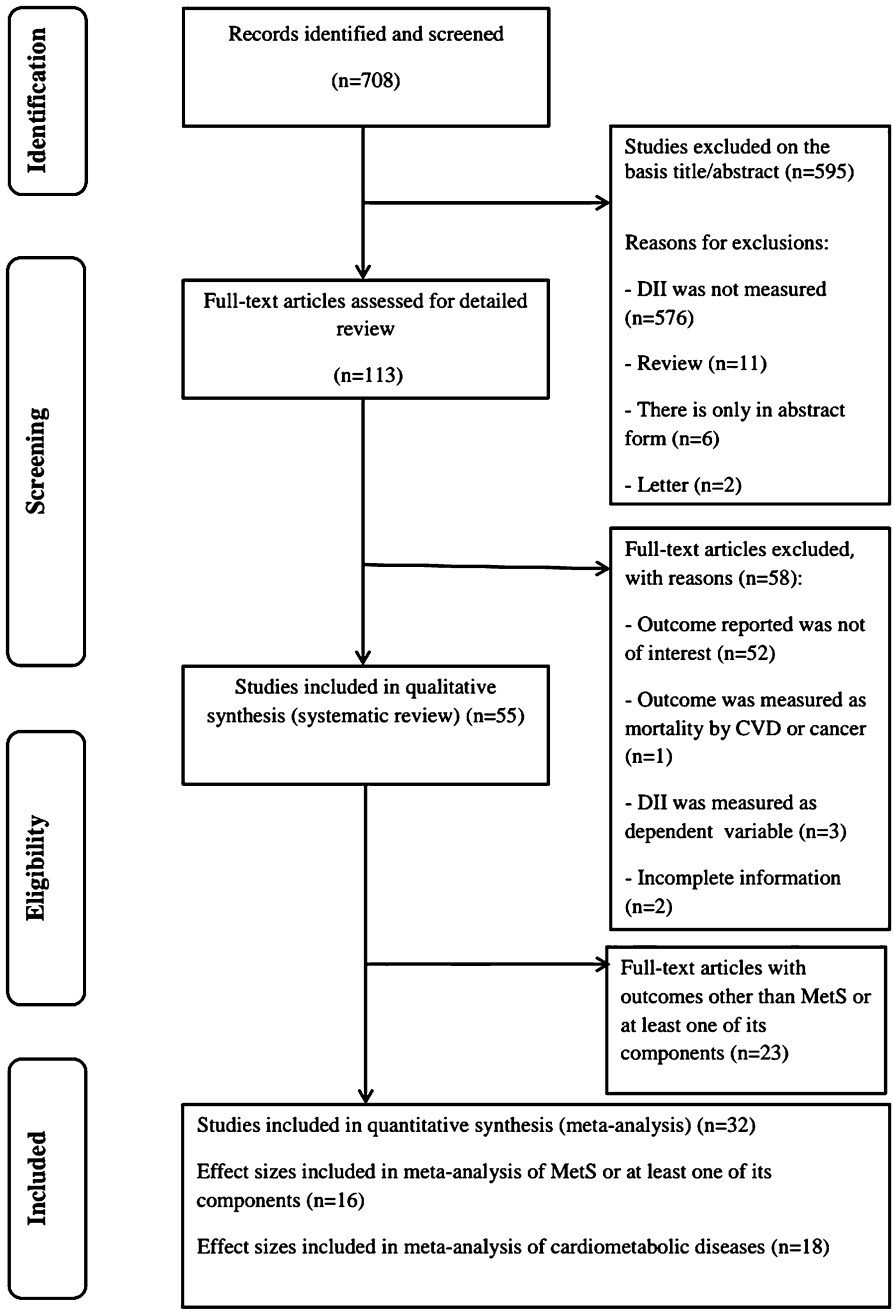
Flow chart of study selection process

A flow diagram for the process of study selection is shown in Fig. 1. The initial search recognized 1,535 papers, and 708 of them remained after duplicate exclusion. Then 653 papers were removed after examining title/abstract and full text of records. The papers were investigated according to the inclusion and exclusion criteria. Eventually, 55 studies were included in the systematic review [15–17, 19–32, 42–79] and 32 records (16 records for CMRFs [17, 19, 20, 23–26, 28, 58, 61, 62, 68, 70, 72, 73, 76] and 18 records for CMDs [15–17, 19, 29–32, 51–57, 77–79]) were selected for meta-analysis. Two studies addressed the association between the DII score and both CMRFs and CMDs outcomes [17, 19]. Due to various outcomes of CMRFs, we considered only studies reporting OR along with 95% confidence interval (CI) for MetS or its components in the meta-analysis.

### Study characteristics

Overall, 55 eligible publications were included in the study. Tables 2 and 3 show the general characteristics of included studies. In general, nine and 10 surveys had considered the morbidity [15–17, 19, 30, 32, 52, 53, 57] (the range of HR was 0.98 [32] to 2.03 [17]) and mortality [29–31, 51, 54–56, 77–79] (the range of HR was 0.98 [51] to 2.50 [78]) of CMDs as outcome, respectively. In addition, 39 studies addressed the association between the DII score and CMRFs [17, 19–28, 42–50, 58–76]. Four case–control studies [15, 21, 57, 74], 23 cohort studies [16, 17, 22–24, 27, 29–32, 44, 45, 48, 51–56, 59, 77–79], and 28 cross-sectional studies [19, 20, 25, 26, 28, 42, 43, 46, 47, 49, 50, 58, 60–73, 75, 76] were included. The number of subjects included in the studies ranged from 90 [48] to 83,054 [79]. The age range of participants was 3–97 years. All records were published between 2014 and 2019. The included studies were conducted in Sweden [15, 45, 55], Australia [24, 29, 32, 53, 77], USA [19, 20, 27, 44, 46, 50, 51, 54, 56, 70, 78, 79], France [23, 52], Spain [16, 17, 22, 47, 49, 63, 69], Germany [31], Italy [57], England [30] Luxembourg [26, 42], Iran [21, 43, 58, 60, 65, 71, 73, 74], Lebanon [25] Korea [68, 75], Poland [28], Myanmar [72], Ireland [62], China [76], Mexico [64], Indonesia [66], Pakistan [67], Brazil [59, 61], and Colombia [48]. The maximum duration of follow up in cohort studies was 25.8 years [31]. Of total included studies, eleven studies were performed on women [24, 27, 29, 32, 50, 54, 55, 59, 72, 74, 75] three on men [31, 53, 67] and 41 reports contained both men and women [15–17, 19–25, 28, 30, 42–49, 51, 52, 56–58, 60–66, 68–71, 73, 76–79]. Validated food frequency questionnaire (FFQ) was applied to assess dietary intakes in 36 studies [15–17, 20–22, 24–30, 32, 42–45, 47, 49, 50, 53–55, 57, 58, 60–62, 64–66, 71, 73, 74, 77, 79], 24-h recall in 13 surveys [16, 19, 46, 51, 56, 59, 67–70, 75, 76, 78], 72- hour recall in one study [63], 24-h recall and FFQ in one report [72] and record in four studies [23, 31, 48, 52]. The exposure variable was considered categorical in 42 studies [15–17, 19–26, 28–32, 43, 46, 48, 51–58, 60, 62, 64, 65, 67, 68, 70, 72–79] and continuous in 32 studies [16, 19, 21, 28–32, 42–45, 47, 49, 50, 52, 54–61, 63, 66, 69, 71, 74, 76, 78, 79].

**Table 2:** Characteristics of studies examined the association of Dietary Inflammatory Index with cardiometabolic diseases

Reference	First author (year)	Study design	Country	Age range/mean age	Gender	Sample size	Diet assessment tool	Number of subjects with CMDs	Duration of follow-up (years)	Number of used dietary factors in DII calculation	Outcome variable	Measure of outcome		Comparison	Type of DII variable (categorical/continuous)	Type of effect size measure		Effect size measure (95% CI)	Study quality	Confounders
15	Bodén et al. 2017	Case–control	Sweden	30–73	F/M	6944 F (NR) M (NR)	FFQ	1389	6.4	30	MI	Morbidity		Quartile 4 (NR) vs. Quartile 1 (NR)	Categorical	OR		1.37 (1.07, 1.73)	8	1, 2, 3, 4, 5, 6, 7, 8
29	Bondonno et al. 2017	Cohort	Western Australia	≥ 70	F	1304	FFQ	269	15	31	ASVD		Mortality	Quartile 4 (1.72, 5.80) vs. Quartile 1 (− 6.14, − 1.37)	Categorical		HR	2.02 (1.30, 3.13)	8	1, 2, 7, 9, 10, 11, 12, 13, 14, 15, 16
														–	Continues (per one SD (2.13 units))			1.36 (1.15, 1.60)		
								150			IHD			Quartile 4 (1.72, 5.80) vs. Quartile 1 (− 6.14, − 1.37)	Categorical			2.51 (1.37, 4.62)		
														–	Continues (per one SD (2.13 units))			1.40 (1.13, 1.75)		
								107			Ischaemic cerebrovascular disease			Quartile 4 (1.72, 5.80) vs. Quartile 1 (− 6.14, − 1.37)	Categorical			1.76 (0.92, 3.40)		
														–	Continues (per one SD (2.13 units))			1.30 (1.00, 1.69)		
51^1^	Deng et al. 2017	Cohort	USA	20–90	F/M	9631 F (5164) M (4467)	24-h dietary recall	676	18	27	CVD	Mortality		Tertile 3 (> 2.0) vs. Tertile 1 (< − 0.20)	Categorical		HR	1.52 (1.18, 1.96)	9	2, 3, 4, 7, 9, 17, 18, 19
						2681 F (1,264) M (1417)		412										1.44 (1.02, 2.04)		
						968 F (451) M (517)		240										0.98 (0.57, 1.67)		
16	Garcia-Arellano et al. 2015	Cohort	Spain	67.0	F/M	7216	FFQ	277	Median follow-up of 4.8	32	CVD	Morbidity		Quartile 4 (median = 1.17) vs. Quartile 1 (median = − 2.46)	Categorical		HR	1.73 (1.15, 2.60)	7	1, 3, 4, 6, 7, 9, 17, 20, 21, 22, 23, 24, 25, 26
														–	Continues (per one SD)			1.22 (1.06, 1.40)		
52	Neufcourt et al. 2016	Cohort	France	35–60	F/M	7743 F (4546) M (3197)	At least 3 valid 24-h dietary records	292	13	36	Overall CVD	Morbidity		Quartile 4 (mean(IQR) (3.1 (1.3)) vs. Quartile 1 (− 1.7 (1.1))	Categorical		HR	1.16 (0.79, 1.69)	7	1, 2, 3, 7, 9, 17, 25, 27, 28, 29, 30
														–	Continues (per one unit)			1.03 (0.96, 1.11)		
								93			MI			Quartile 4 (mean(IQR) (3.1 (1.3)) vs. Quartile 1 (− 1.7 (1.1))	Categorical			2.26 (1.08, 4.71)		
														–	Continues (per one unit)			1.12 (0.98, 1.28)		
														Quartile 4 (mean (IQR)2.41 (1.00))vs. Quartile 1 (− 1.86 (1.20))	Categorical			1.62 (0.88, 2.97)		
														–	Continues (per one unit)			1.12 (0.98, 1.27)		
								58			Stroke			Quartile 4 (mean (IQR) (3.1 (1.3))vs. Quartile 1 (− 1.7 (1.1))	Categorical			1.22 (0.56, 2.65)		
														–	Continues (per one unit)			1.05 (0.89, 1.24)		
								128			AP/RI			Quartile 4 (mean (IQR) (3.1 (1.3)) vs. Quartile 1 (− 1.7 (1.1))	Categorical			0.73 (0.41, 1.30)		
														–	Continuous (per one unit)			0.97 (0.87, 1.09)		
								13			Sudden deaths			Quartile 4 (mean (IQR) (3.1 (1.3))vs. Quartile 1 (− 1.7 (1.1))	Categorical			NR		NR
														–	Continuous (per one unit)					
53	O’Neil et al. 2015	Cohort	Australia	20–97	M	1363	FFQ	76	5	22	CVD	Morbidity		Pro-inflammatory (positive DII) vs. anti- inflammatory (negative DII)	Categorical		OR	2.00 (1.01, 3.96)	7	1, 3, 4, 6, 7, 9, 31, 32
17	Ramallal et al. 2015	Cohort	Spain	38	F/M	18,794 F (NR) M (NR)	FFQ	117	Median (8.9)	28	CVD	Morbidity		Quartile 4 (− 0.74, 3.97) vs. Quartile 1 (− 5.14, − 2.68)	Categorical		HR	2.03 (1.06, 3.88)	7	1, 2, 3, 6, 7, 9, 17, 22, 23, 24, 25, 33, 34, 35, 36
54	Shivappa et al. 2016	Cohort	USA	55–69	F	28,677	FFQ	6528	Mean ± SD (20.7 ± 7.0)	NR	CVD	Mortality		Quartile 4 (0.64, 4.65) vs. Quartile 1 (− 5.75, − 2.50)	Categorical		HR	1.09 (1.01, 1.18)	8	1, 2, 6, 7, 9, 22, 25, 33, 37, 38
														–	Continuous (per one unit)			1.04 (1.01, 1.07)		
								3381			CHD			Quartile 4 (0.64, 4.65) vs. Quartile 1 (− 5.75, − 2.50)	Categorical			1.17 (1.05, 1.30)		
														–	Continuous (per one unit)			1.07 (1.03, 1.11)		
								1439			Stroke			Quartile 4 (0.64, 4.65) vs. Quartile 1 (− 5.75, − 2.50)	Categorical			1.04 (0.88, 1.22)		
														–	Continuous (per one unit)			1.01 (0.95, 1.08)		
								417	0–4.99		CVD							1.06 (0.95, 1.19)		
								736	5–9.99									1.14 (1.05, 1.24)		
								1177	10.00–14.99									1.01 (0.94, 1.07)		
								1825	15.00–19.99									1.07 (1.01, 1.13)		
								2373	20.00–25.00									1.00 (0.96, 1.05)		
								260	0–4.99		CHD							1.13 (0.68, 1.31)		
								447	5–9.99									1.15 (1.03, 1.28)		
								681	10.00–14.99)									0.98 (0.90, 1.07)		
								918	15.00–19.99									1.12 (1.04–1.20)		
								1075	20.00–25.00									1.03 (0.96, 1.11)		
								54	0–4.99		Stroke							1.05 (0.77, 1.42)		
								129	5–9.99									1.07 (0.87, 1.32)		
								233	10.00–14.99)									1.06 (0.92, 1.23)		
								441	15.00–19.99									1.04 (0.93, 1.16)		
								582	20.00–25.00									0.96 (0.87, 1.06)		
55	Shivappa et al. 2016	Cohort	Sweden	NR	F	33,747	FFQ	2399	15	27	CVD	Mortality		Quintile 5 (≥ 1.91) vs. Quintile 1 (≤ − 0.67)	Categorical		HR	1.26 (0.93, 1.70)	8	1, 2, 3, 7, 9, 25, 39
														–	Continues (per one unit)			1.04 (0.98, 1.12)		
30	Shivappa et al. 2017	Cohort	Germany	45–64	M	1297	7-day dietary record	244	Survey1: median follow-up = 25.8 Survey 3: median follow-up = 16.7	NR	CVD	Mortality		Quartile 4 (median (2.507)) vs. Quartile 1 (median (− 0.803))	Categorical		HR	1.19 (0.76, 1.86)	7	1, 2, 3, 6, 7, 9, 22, 25, 40, 41, 42
														–	Continues (per one unit)			1.05 (0.92, 1.20)		
								155			CHD			Quartile 4 (median (2.507)) vs. Quartile 1 (median (− 0.803))	Categorical			1.02 (0.57, 1.82)		
														–	Continues (per one unit)			1.01 (0.86, 1.18)		
						1252		213			CHD	Morbidity		Quartile 4 (median (2.507)) vs. Quartile 1 (median (− 0.803))	Categorical			1.53 (0.93, 2.53)		
														–	Continues (per one unit)			1.11 (0.97, 1.27)		
56	Shivappa et al. 2017	Cohort	USA	> 19	F/M	12,366 F (NR) M (NR)	One in-person 24-h dietary recall	1235	Mean ± SD (13.5 ± 4.0)	27	CVD	Mortality		Tertile 3 (2.03, 4.83) vs. Tertile 1 (− 5.60, − 0.22)	Categorical		HR	1.46 (1.18, 1.81)	8	2, 3, 6, 7, 9, 17, 18, 22, 33, 43
														–	Continuous (1-unit increment in DII (corresponding to 0.5 standard deviation increase))			1.06 (1.02, 1.09)		
57	Shivappa et al. 2017	Case–control	Italy	Case (19–79) Control (16–79)	F/M	1442 F (423) M (1019)	FFQ	760	–	30	AMI	Morbidity		Quartile 4 (1.10, 5.45) vs. Quartile 1 (− 4.46, − 1.38)	Categorical		OR	1.60 (1.06, 2.41)	7	1, 2, 3, 6, 7, 9, 17, 22, 23, 24, 25, 44
														–	Continuous (one unit increase in DII equals to ~ 9% range of DII in this study (− 6.22 to + 5.45)			1.14 (1.05, 1.24)		
31	Vissers et al. 2016	Cohort	Australia	52 (1)	F	6,972	FFQ	335	Mean ± SD (11 ± 1.6)	25	CVD	Morbidity		(DII ≥ 0) vs. (DII < 0)	Categorical		HR	1.03 (0.76, 1.42)	8	1, 2, 3, 6, 7, 9, 22, 25, 37, 39, 45
														–	Continuous (per one SD)			0.98 (0.84, 1.15)		
								191			IHD			(DII ≥ 0) vs. (DII < 0)	Categorical			1.33 (0.86, 2.06)		1, 6, 7, 9, 22
														–	Continuous (per one SD)			1.08 (0.88, 1.33)		
								69			MI			(DII ≥ 0) vs. (DII < 0)	Categorical			1.59 (0.72, 3.52)		
														–	Continuous (per one SD)			1.40 (0.97, 2.01)		
								59			Cerebrovascular disease			(DII ≥ 0) vs. (DII < 0)	Categorical			0.57 (0.29, 1.15)		
														–	Continuous (per one SD)			0.72 (0.50, 1.02)		
								40			Stroke			(DII ≥ 0) vs. (DII < 0)	Categorical			0.55 (0.24, 1.26)		
														–	Continuous (per one SD)			0.77 (0.51, 1.18)		
19	Wirth et al. 2016	Cross-sectional	USA	20–80	F/M	15,613 F (8047) M (7566)	24-h dietary recall	1734	–	27	Combined circulatory disorders	Morbidity		Quartile 4 (1.94, 4.83) vs. Quartile 1 (− 5.81, − 0.81)	Categorical		POR	1.30 (1.06, 1.58)	5	2, 7, 9, 46
														–	Continuous (per one unit)			1.05 (1.01, 1.08)		
						15,622		501			Congestive heart failure			Quartile 4 (1.94, 4.83) vs. Quartile 1 (− 5.81, − 0.81)	Categorical			1.38 (1.09, 1.74)		
														–	Continuous (per one unit)			1.06 (1.02, 1.10)		
						15,623		634			CHD			Quartile 4 (1.94, 4.83) vs. Quartile 1 (− 5.81, − 0.81)	Categorical			0.96 (0.72, 1.28)		
														–	Continuous (per one unit)			0.99 (0.94, 1.05)		
						15,643		423			AP			Quartile 4 (1.94, 4.83) vs. Quartile 1 (− 5.81, − 0.81)	Categorical			0.83 (0.54, 1.28)		
														–	Continuous (per one unit)			0.95 (0.89, 1.02)		
						15,664		685			Heart Attack			Quartile 4 (1.94, 4.83) vs. Quartile 1 (− 5.81, − 0.81)	Categorical			1.48 (1.12, 1.97)		
														–	Continuous (per one unit)			1.06 (1.01, 1.12)		
						15,666		604			Stroke			Quartile 4 (1.94, 4.83) vs. Quartile 1 (− 5.81, − 0.81)	Categorical			1.56 (1.21, 2.01)		
														–	Continuous (per one unit)			1.09 (1.04, 1.15)		
32	Shivappa et al. 2017	Cohort	England	35–55	F/M	7627 F (2319) M (5308)	FFQ	264	22	27	CVD	Mortality		Tertile 3 (0.74–3.82) vs. Tertile 1 (− 3.08–0.39)	Categorical		HR	1·46 (1·00, 2·13)	7	1, 2, 3, 6, 9, 7, 17, 18, 22, 29, 33, 39, 47, 48, 49, 50
														–	Continuous (per one SD (1.3 units))			1.23 (1.04, 1.47)		
77	Hodge et al. 2018	Cohort	Australia	40–69	F/M	39,532 F (16,051) M (23,481)	FFQ	2,081	19	29	CVD	Mortality		Quintile 5 (0.7, 4.9) vs. Quintile 1 (− 5.0, − 2.4)	Categorical		HR	1.16 (1.01, 1.33)	8	6, 17, 24, 33, 39, 51, 52
78	Mark Park et al. 2018	Cohort	USA	20–90	F/M	3733 F (1553) M (2180)	24-h dietary recall	252	18.5	27	CVD	Mortality		Tertile 3 (1.97, 4.55) vs. Tertile1 (− 5.08, − 0.24)	Categorical		HR	2.50 (1.60, 3.91)	8	2, 3, 7, 9, 17, 18, 25, 53
														–	Continuous (per one SD)			1.32 (1.10, 1.58)		
79	Park et al. 2018	Cohort	USA	45–75	F	83,054	FFQ	7811	18.2 ± 4.9	28	CVD	Mortality		Quintile 5 (− 0.06, 4.95) vs. Quintile 1) − 6.64, − 3.91)	Categorical		HR	1.29 (1.17–1.42)	8	1, 2, 3, 6, 7, 9, 18, 25, 29, 37, 39
														–	Continues (per one unit)			1.04 (1.03,1.06)		
					M	67,351		8401						Quintile 5 (− 0.06, 4.95) vs. Quintile 1 (− 6.64, − 3.91)	Categorical			1.13 (1.03, 1.23)		
														–	Continues (per one unit)			1.03 (1.01, 1.04)		

1—total energy intake, 2—body mass index, 3—physical activity, 4—systolic blood pressure, 5—total cholesterol, 6—diabetes, 7—smoking, 8—postsecondary academic education, 9—age, 10—energy expended in physical activity, 11—socioeconomic status, 12—use of low-dose aspirin, 13—use of antihypertensive medication, 14—use of statins, 15—prevalent atherosclerotic vascular disease, 16—treatment code, 17—sex, 18—race, 19—HbA1c, 20—overweight/obesity, 21—waist to height ratio, 22—hypertension, 23—dyslipidemia, 24—family history of premature cardiovascular disease, 25—educational level, 26—stratified by intervention group and center, 27—supplementation, 28—number of 24-h records, 29—marital status, 30—treatment allocation group (placebo or active), 31—diastolic blood pressure, 32—waist circumference, 33—previous history of other cardiovascular diseases, 34—following a special diet, 35—hours spent sitting down, 36—hours spent watching television, 37—hormone replacement therapy use, 38—prevalent cancer (yes/no), 39—alcohol intake, 40—survey number, 41—place of residence, 42—ratio of total cholesterol and high density lipoprotein cholesterol, 43—poverty index, 44—coffee consumption, 45—menopausal status, 46—family member, 47—occupational grade, 48—use of lipid-lowering drugs, 49—high density lipoprotein cholesterol, 50—longstanding illness, 51—country of birth, 52—socio-economic indexes for areas quintile, 53—income

*F* female, *M* male, *FFQ* food frequency questionnaire, *MI* myocardial infarction, *AMI* acute myocardial infarction, *ASVD* atherosclerotic vascular disease, *IHD* ischaemic heart disease, *CVD* cardiovascular diseases, *AP/RI* angina pectoris/revascularization intervention, *CHD* coronary heart disease, *OR* odds ratio, *POR* prevalence odds ratio, *HR* hazard ratio, *NR* not reported

^1^Participants included three groups of normal, pre-diabetic and diabetic adults

**Table 3:** Characteristics of studies examined the association of dietary inflammatory index with cardio-metabolic risk factors

Reference	First author (year)	Study design	Country	Age range/mean age	Gender	Sample size	Diet assessment tool	The number of subjects with CMRFs	Duration follow-up (years)	Number of used dietary factors in DII calculation	Outcome variable	Measure of outcome	Comparison		Type of DII variable (categorical/ continuous)	Type of effect size measure	Effect size measure (95% CI)	Study quality	Confounders
24	Alkerwi et al. 2014	Cross-sectional	Luxembourg	18–69	F/M	1352 F (695) M (657)	FFQ	430	–	24	Abdominal obesity	Morbidity	DII > 1 vs. DII ≤ 1		Categorical	OR	1.12 (0.81, 1.56)	7	3, 7, 9, 11, 17, 25
								249			Low HDL-C						1.46 (1.00, 2.13)		
								351			Hyper- triglyceridemia						1.17 (0.82, 1.67)		
								741			HTN						0.85 (0.61, 1.18)		
								307			Hyperglycemia						1.30 (0.90, 1.89)		
								346			MetS						1.18 (0.81, 1.71)		
42	Alkerwi et al. 2015	Cross-sectional	Luxembourg	18–69	F/M	1040 F (NR) M (NR)	FFQ	1040	-	NR	HDL-C (mmol/l)	Morbidity	–		Continuous (each 1-z score difference across the DII)	β -Coefficient	0	8	3, 7, 9, 17, 25
											TC (mmol/l)						0.0409		
											TG (mmol/l)						− 0.00003		
											LDL-C (mmol/l)						0.0003		
											ApoA1 (mg/l)						0.02		
											Apo B (mg/l)						0.13		
						1106 F (NR) M (NR)		1,106			FBS (mmol/l)						− 0.0002		
											HbA1c (%)						− 0.0001		
											HOMA-IR						− 0.017		
											Insulin (mg/l)						− 0.22		
						1153 F (NR) M (NR)		1153			BMI (kg/m^2^)						− 0.003		
											WC (cm)						0.002		
						1007 F (NR) M (NR)		1007			SBP (mmHg)						− 0.001		
											DBP (mmHg)						0.587		
43	Moslehi et al. 2016	Cross-sectional	Iran	19–75	F/M	2975 F (1,641) M (1304)	FFQ	1,007	–	37	Glucose tolerance abnormality	Morbidity	Quartile 4 (0.29, 5.23) vs. Quartile 1 (− 5.82, − 2.67)		Categorical	OR	1.15 (0.90, 1.48)	8	2, 3, 6, 7, 9, 17, 22, 48
								590			IFG						1.09 (0.83, 1.44)		
								259			IGT						1.24 (0.84, 1.81)		
								286			Type 2 diabetes						0.98 (0.66, 1.47)		
								1923			Insulin resistance						1.18 (0.91, 1.51)		2, 3, 6, 7, 9, 17, 22, 48, 54
								2975			FBS levels (mmol/L)		-		Continuous	β-Coefficient	0.01		
											Postload glucose levels (mmol/L)						0.04		
											Fasting insulin (U/mL)						0.02		
											HOMA-IR						0.02		
											HOMA-B						0.01		
											QUICKI						− 0.02		
25	Naja et al. 2017	Cross-sectional	Lebanon	> 18	F/M	330 F (NR) M (NR)	FFQ	171	–	25	Abdominal obesity	Morbidity	Quintile 5 (NR) vs. Quintile 1 (NR)		Categorical	OR	0.66 (0.29, 1.48)	7	3, 7, 9, 17, 25, 29, 55
								105			Low HDL-C						0.74 (0.31, 1.75)		
								103			Hyper- triglyceridemia						0.84 (0.35, 1.03)		
						329		132			HTN						0.40 (0.23, 1.04)		
						331		151			Hyperglycemia						1.80 (0.80, 4.01)		
						328		114			MetS						0.72 (0.31, 1.67)		
23	Neufcourt et al.. 2015	Cohort	France	35–60	F/M	3726 F (2367) M (1359)	At least 3 valid 24-h dietary records	524	13	36	MetS	Morbidity	Quartile 4 (mean (IQR) 2.97 (1.27)) vs. Quartile 1 (− 1.76 (1.07))		Categorical	OR	1.39 (1.01,1.92)	7	1, 3, 7, 9, 17, 25, 56
22	Ramallal et al.. 2017	Cohort	Spain	37.4	F/M	7027 F (4535) M (2492)	FFQ	1433 overweight (1409) Obese (24)	10	28	Overweight/Obesity	Morbidity	Quartile 4 (− 0.59, 4) vs. Quartile 1 (− 5.1, -2.5)		Categorical	HR	1.32 (1.08, 1.60)	8	1, 2, 3, 7, 9, 17, 34, 35, 36, 39, 57, 58, 59, 60
17	Ramallal et al. 2015	Cohort	Spain	38	F/M	18,794 F (NR) M (NR)	FFQ	NR	2	28	HTN	Morbidity	Quartile 4 (− 0.74, 3.97) vs. Quartile 1 (− 5.14, − 2.68)		Categorical	OR	1.71 (1.11, 2.64)	7	1, 2, 3, 7, 9, 17, 24, 25, 35, 36, 39, 57
											Hypercholesterolemia						1.04 (0.69, 1.57)		
44	Sen et al. 2018	Cohort	USA	2.8- 4.9	F/M	922 F (NR) M (NR)	FFQ	922	4.5	NR	BMI z-score	Morbidity	-		Continues (per 1 point increment in pregnancy DII)	β-Coefficient	Girls: 0.04 (− 0.09, 0.17) Boys: 0.16 (0.02, 0.29)	8	9, 17, 25, 18, 53
						775		775			FFM index (kg/m^2^)						Girls: 0.06 (− 0.13, 0.24) Boys: 0.19 (− 0.01, 0.39)		
											FM index (kg/m^2^)						Girls: 0.14 (− 0.13, 0.40) Boys: 0.13 (− 0.14, 0.41)		
											Trunk fat mass index (kg/m^2^)						Girls: 0.06 (− 0.05, 0.18) Boys: 0.06 (− 0.06, 0.19)		
						922		922			WC (cm)						Girls: 0.21 (− 0.77, 1.19) Boys: 0.93 (− 0.07, 1.92)		
											SS + Tr (mm)						Girls: 0.31 (− 0.92, 1.53) Boys: 1.12 (0.01, 2.23)		
											Fasting insulin (uU/ml)						Girls: -0.06 (− 1.10, 0.97) Boys: − 0.80 (− 1.85, 0.24)		
											LDL-C (mg/dl)						Girls: − 0.17 (− 4.11, 3.77) Boys: − 0.80 (− 1.85, 0.24)		
						481		481			Mid-childhood metabolic risk score						Girls: 0.04 (− 0.08, 0.16) Boys: 0.02 (− 0.10, 0.14)		
28	Sokol et al. 2016	Cross-sectional	Poland	45–64	F/M	3862 F (2572) M (1290)	FFQ	1759	–	22	Abdominal obesity	Morbidity	Quartile 4 (− 0.75, 4.00) vs. Quartile 1 (− 4.56,− -2.62)		Categorical	OR	0.79 (0.61, 1.03)	7	2, 9
															Continuous (per one unit)		0.95 (0.89, 1.02)		
								615			Low HDL-C				Categorical		0.62 (0.48, 0.80)		
															Continuous (per one unit)		0.89 (0.84, 0.95)		
								815			Hyper- triglyceridemia				Categorical		1.04 (0.84, 1.30)		
															Continuous (per one unit)		1.01 (0.95, 1.07)		
								2590			HTN				Categorical		1.05 (0.86, 1.28)		
															Continuous (per one unit)		1.02 (0.97, 1.07)		
								1402			Hyperglycemia				Categorical		1.11 (0.91, 1.34)		
															Continuous (per one unit)		1.04 (0.99, 1.09)		
								1159			MetS				Categorical		0.96 (0.77, 1.19)		
															Continuous (per one unit)		0.99 (0.94, 1.05)		
21	Vahid et al. 2016	Case–control	Iran	31–67	F/M	414 F (NR) M (NR)	FFQ	214	-	27	Pre-diabetes	Morbidity	Tertile 3 (> − 0.54) vs. Tertile 1 (< − 1.21)		Categorical	OR	18.88 (7.02, 50.82)	7	1, 2, 3, 7, 9, 17, 25
													-		Continuous (per one unit)		3.62 (2.50, 5.22)		
								414			FBS levels (mmol/l)		Tertile 3 (> − 0.54) vs. Tertile 1 (< − 1.21)		Categorical	β-Coefficient	4.49 (1.89, 7.09)		2, 6, 7, 9, 25, 39, 61, 62
													-		Continuous (per one tertile)		2.18 (1.21, 3.15)		
								414			OGTT (mg/dl)		Tertile 3 (> − 0.54) vs. Tertile 1 (< − 1.21)		Categorical		8.76 (1.78, 15.73)		
													–		Continu ous (per one tertile)		4.08 (1.45, 6.71)		
								414			HbA1C (mmol/l)		Tertile 3 (> − 0.54) vs. Tertile 1 (<− -1.21)		Categorical		0.30 (0.17, 0.42)		
													-		Continuous (per one tertile)		0.12 (0.07, 0.17)		
								414			HDL-C (mg/dl)		Tertile 3 (> − 0.54) vs. Tertile 1 (< − 1.21)		Categorical		− 3.39 (− 5.94, − 0.84)		
													–		Continuous (per one tertile)		− 1.10 (− 2.06, − 0.13)		
								414			LDL-C (mg/dl)		Tertile 3 (>− 0.54) vs. Tertile 1 (<− 1.21)		Categorical		16.37 (11.04, 21.69)		
													-		Continuous (per one tertile)		5.51 (3.47, 7.54)		
								414			TG (mg/dl)		Tertile 3 (> − 0.54) vs. Tertile 1 (< − 1.21)		Categorical		21.01 (8.61, 33.42)		
															Continuous (per one tertile)		12.66 (8.06, 17.27)		
								414			LBM (%)		Tertile 3 (> − 0.54) vs. Tertile 1 (< − 1.21)		Categorical		− 3.11 (− 4.83, − 1.39)		
													-		Continuous (per one tertile)		− 1.26 (− 1.91, − 0.61)		
								414			Body fat (%)		Tertile 3 (> − 0.54) vs. Tertile 1 (< − 1.21)		Categorical		2.41 (0.56, 4.26)		
													-		Continuous (per one tertile)		1.10 (0.40, 1.79)		
26	Vissers et al. 2017	Cohort	Australia	52	F	7,169	FFQ	1680	12	25	HTN	Morbidity	DII ≥ 0 vs. DII < 0		Categorical	OR	1.24 (1.06, 1.45)	7	1, 2, 3, 6, 7, 9, 25, 40, 45
													-		Continuous (per 1 SD increase in DII score)		1.07 (0.99, 1.15)		
	Michael D. Wirth et al. 2014	Cross-sectional	United states of America	42.4 ± 8.5	F/M	447 F (112) M (335)	FFQ	125	-	DII (36 food items)	MetS	Morbidity	Presence of at least three of these components WC of ≥ 102 cm for males or ≥ 88 for females; BP ≥ 130 for systolic or ≥ 85 for diastolic or reported diagno sed hypertension or antihypertensive medication; HDL-C of < 40 mg/dL in men and < 50 in women; TG ≥ 150 mg/dL, and glucose ≥ 100 mg/dL or reported treatment for diabetes	Quartile 4 (2.64, 5.89) vs. Quartile 1 (− 6.27, − 1.26)	Categorical	OR	0.87 (0.46–1.63)		Age, sex
20	Wirth et al. 2014	Cross-sectional	USA	42.4	F/M	447 F (112) M (335)	FFQ	150	-	36	Abdominal obesity	Morbidity	Quartile 4 (2.64, 5.89) vs. Quartile 1 (− 6.27, − 1.26)		Categorical	OR	0.93 (0.52, 1.67)	5	14, 64
						444		185			Low HDL-C						1.03 (0.59, 1.83)		17, 18, 39
						444		136			Hyper-triglyceridemia						0.77 (0.42, 1.42)		17, 18
						447		181			HTN						1.14 (0.64, 2.02)		9, 17, 39
						445		115			Hyperglycemia						2.03 (1.08, 3.82)		9, 17
						444		125			MetS						0.87 (0.46, 1.63)		
46	Tyrovolas et al. 2017	Cross-sectional	USA	≥ 20	F/M	7880 F (NR) M (NR)	24-h dietary recall	NR	–	27	CVD-RF morbidity index (included obesity, diabetes, hypertension, and hypercholesterolemia. The total number of these risk factors was calculated (range 0–4) for each individual and used as the outcome)	Morbidity	Quartile 4 (NR) vs. Quartile 1 (NR)		Categorical	OR	1.39 (1.15, 1.67)	8	3, 7, 9, 17, 18, 25, 29, 33, 59
													–		Continuous (per one unit)		1.07 (1.03, 1.10)		
19	Wirth et al. 2016	Cross-sectional	USA	20–80	F/M	15,666 F (NR) M (NR)	24-dietary recall	5408	-	27	HTN	Morbidity	Quartile 4 (1.94, 4.83) vs. Quartile 1 (-5.81, -0.81)		Categorical	POR	1.19 (1.05, 1.34)	5	2, 7, 9, 46
													-		Continuous (per one unit)		1.04 (1.01, 1.06)		
27	Sen et al. 2015	Cohort	USA	32.2	F	1779	FFQ	160	6 months	28	Isolated hyperglycemia	Morbidity	-		Continuous (per one unit)	OR	0.94 (0.82, 1.07)	7	2, 7, 9, 18, 25, 53
								58			Impaired glucose tolerance						0.88 (0.71, 1.09)		
								96			GDM						0.78 (0.65, 0.95)		
						1775		NR			Inadequate pregnancy weight gain						0.97 (0.86, 1.09)		
											Excessive pregnancy weight gain						0.95 (0.87, 1.03)		
						1772		25			Chronic HTN						0.91 (0.65, 1.28)		
								122			Gestational HTN						0.97 (0.83, 1.14)		
								62			Preeclampsia						1.04 (0.85, 1.26)		
47	Ruiz-Canela et al. 2015	Cross-sectional	Spain	56–80	F	4145	FFQ	4145	–	33	BMI (kg/m^2^)	Morbidity	–		Continues	Pearson coefficient (r)	0.06 (0.03, 0.09)	8	1, 3, 6, 7, 9, 22, 25, 29,
											WC (cm)						0.05 (0.02, 0.08)		
											WHtR (%)						0.06 (0.03, 0.09)		
				55–80	M	3091		3091			BMI (kg/m^2^)						0.05 (0.01, 0.08)		
											WC (cm)						0.08 (0.05, 0.20)		
											WHtR (%)						0.09 (0.06, 0.13)		
48	Camargo-Ramos et al. 2017	Cohort	Colombia	39.7	F/M	90 F (NR) M (NR)	24-dietary record	90	NR	28	DXA total tissue (% fat)	Morbidity	–		Categorical (Anti-Inflammatory Diet (− 3.71 to − 0.37) and Inflammatory Diet (0.13–3.64))	Pearson coefficient (r)	Anti-inflammatory diet = − 0.122, pro-inflammatory diet = 0.111	7	9, 17
											TC (mg/dL)						Anti-inflammatory diet = -0.210, Pro-inflammatory diet = 0.010		
											TG (mg/dL)						Anti-inflammatory diet = − 0.354, pro-inflammatory diet = − 0.009		
											HDL-C (mg/dL)						Anti-inflammatory diet = − 0.100, Pro-inflammatory diet = 0.028		
											LDL-C (mg/dL)						Anti-Inflammatory Diet = 0.350, Pro-Inflammatory Diet = -0.084		
											FBS (mg/dL)						Anti-inflammatory diet = − 0.422, pro-inflammatory diet = − 0.228		
											MetScore						Anti-inflammatory diet = − 0.282, pro-inflammatory diet = 0.410		
											HbAc1 (%)						Anti-inflammatory diet = 0.004, pro-inflammatory diet = 0.090		
											FMD (%)						Anti-inflammatory diet = 0.261, pro-inflammatory diet = − 0.233		
											PWV (m/s)						Anti-inflammatory diet =− 0.437, pro-inflammatory diet = 0.014		
											Aortic SBP (mm Hg)						Anti-inflammatory diet =− 0.271 pro-inflammatory diet = − 0.126		
											Aortic pulse pressure (mm Hg)						Anti-inflammatory diet =− 0.271, pro-inflammatory diet = − 0.055		
											Brachial augmentation index (%)						Anti-inflammatory diet = − 0.300, pro-inflammatory diet = − 0.209		
											Aortic augmentation index (%)						Anti-inflammatory diet = − 0.299, pro-inflammatory diet = − 0.064		
											MAP (mm Hg)						Anti-inflammatory diet = − 0.011, pro-inflammatory diet = 0.079		
49	Cantero et al. 2017	Cross-sectional	Spain	55–80	F/M	794 F (NR) M (NR)	FFQ	794	–	NR	BMI (kg/m^2^)	Morbidity	–		Continues	Pearson coefficient (r)	0.130	8	NR
50	Tabung et al. 2017	Cross-sectional	USA	25–42	F	3985	FFQ	3985	–	38	Adiponectin	Morbidity	–		Continues	Pearson coefficient (r)	− 0.10	8	NR
				40–75	M	4176		4176									− 0.05		
58	Abdurahman et al. 2018	Cross-sectional	Iran	19–59	F/M	277 F (233) M (44)	FFQ	176	–	32	MUO	Morbidity	Quartile 4 (7.98) vs. Quartile 1 (− 8.87)		Categorical	OR	2.58 (1.19, 5.59)	8	2, 3, 9, 17, 65
													-		Continues (per one quartile)		1.18 (1.01, 1.39)		
								NR			Abdominal obesity		Quartile 4 (7.98) vs. Quartile 1 (− 8.87)		Categorical		0.58 (0.16, 2.05)		
													-		Continues (per one quartile)		0.91 (0.73, 1.14)		
								NR			Low HDL-C		Quartile 4 (7.98) vs. Quartile 1 (− 8.87)		Categorical		1.19 (0.55, 2.57)		
													-		Continues (per one quartile)		1.01 (0.87, 1.18)		
								NR			Hyper- triglyceridemia		Quartile 4 (7.98) vs. Quartile 1 (− 8.87)		Categorical		1.66 (0.82, 3.37)		
													-		Continues (per one quartile)		1.11 (0.95, 1.13)		
								NR			HTN		Quartile 4 (7.98) vs. Quartile 1 (− 8.87)		Categorical		1.66 (0.83, 3.34)		
													–		Continues (per one quartile)		1.11 (0.96, 1.29)		
								NR			Hyperglycemia		Quartile 4 (7.98) vs. Quartile 1 (− 8.87)		Categorical		1.89 (0.92, 3.91)		
													-		Continues (per one quartile)		1.13 (0.97, 1.32)		
59	Andrade et al. 2018	Cohort	Brazil	43.0	F	132	24-h dietary recall	132	0.5	21	Postoperative weight (kg)	Morbidity	-		Continues (Per one unit)	β-coefficient	2.02 (0.33, 3.70)	7	1, 9
											Postoperative body fat mass (kg)						1.78 (0.51, 3.04)		
60	Aslani et al. 2018	Cross-sectional	Iran	6–18	F/M	5427 F (2,541) M (2,886)	FFQ	5427	–	25	BMI z-score	Morbidity	Quartile 4 (1.50 to 4.26) vs. Quartile 1 (− 4.42 to − 1.63)		Categorical	β-coefficient	0.07 (0.01, 0.14)	8	2, 9, 11, 17, 36, 41
													-		Continues (per one quartile)		0.01 (-0.002, 0.04)		
											Wrist Circumference (cm)		Quartile 4 (1.50 to 4.26) vs. Quartile 1 (− 4.42 to − 1.63)		Categorical		0.06 (− 0.09, 0.21)		
													-		Continues (per one quartile)		0.03 (− 0.01, 0.08)		
											NC (cm)		Quartile 4 (1.50 to 4.26) vs. Quartile 1 (− 4.42 to − 1.63)		Categorical		− 0.08 (− 0.43, 0.26)		
													–		Continues (per one quartile)		0.00 (− 0.11, 0.11)		
											WC (cm)		Quartile 4 (1.50 to 4.26) vs. Quartile 1 (− 4.42 to − 1.63)		Categorical		0.89 (0.07, 1.70)		
													-		Continues (per one quartile)		0.27 (0.01, 0.53)		
											HC (cm)		Quartile 4 (1.50 to 4.26) vs. Quartile 1 (− 4.42 to − 1.63)		Categorical		1.13 (0.29, 1.96)		
													-		Continues (per one quartile)		0.39 (0.13, 0.65)		
											WHR		Quartile 4 (1.50 to 4.26) vs. Quartile 1 (− 4.42 to − 1.63)		Categorical		0.00 (− 0.01, 0.01)		
													-		Continues (per one quartile)		− 0.001 (− 0.004, 0.002)		
											WHtR		Quartile 4 (1.50 to 4.26) vs. Quartile 1 (− 4.42 to − 1.63)		Categorical		0.004 (− 0.01, 0.02)		
													-		Continues (per one quartile)		0.002 (− 0.04, 0.009)		
											Parental BMI (kg/m^2^)		Quartile 4 (1.50 to 4.26) vs. Quartile 1 (− 4.42 to − 1.63)		Categorical		1.05 (0.61, 1.49)		
													-		Continues (per one quartile)		0.34 (0.20, 0.48)		
61	Carvalho et al. 2018	Cross-sectional	Brazil	23–25	F	1,034	FFQ	110	–	35	Insulin resistance	Morbidity	-		Continues (per one unit)	PR	0.96 (0.87, 1.07)	7	9, 53
								67			MetS						1.05 (0.91, 1.20)		
					M	942		134			Insulin resistance						0.98 (0.89, 1.08)		
								180			MetS						0.98 (0.91, 1.07)		
62	Phillips et al. 2018	Cross-sectional	Ireland	50–69	F/M	1992 F (1016) M (976)	FFQ	NR	–	26	MetS	Morbidity	< Median DII (− 5.10 to − 1.28) vs > Median DII (− 1.28 to 3.68)		Categorical	OR	1.37 (1.01, 1.88)	8	2, 9, 17, 66
											large VLDL particles (nmol/L)						1.28 (1.07, 1.54)		
											small HDL particle size (nmol/L)						1.45 (1.21, 1.74)		
											small LDL particle size (nmol/L)						1.54 (1.28, 1.84)		
											Lipoprotein Insulin Resistance score						1.24 (1.10, 1.50)		
63	Correa-Rodríguez et al. 2018	Cross-sectional	Spain	18–25	F/M	599 F (414) M (185)	72-h dietary recall	599	–	25	BMI (kg/m^2^)	Morbidity	–		Continues (per one unit)	β-coefficient	− 0.073 (− 0.487, 0.026)	7	1, 9, 17
											FM (kg)						− 0.074 (− 1.052, 0.050)		
											PFM (%)						− 0.047 (0.845, 0.170)		
											FFM (kg)						− 0.059 (− 0.842, − 0.107)		
											VFR						− 0.017 (− 0.217, 0.142)		
64	Denova-Gutiérrez et al. 2018	Cross-sectional	Mexico	20–69	F/M	1174 F (515) M (659)	Semi-quantitative FFQ	201	–	27	T2DM	Morbidity	Quintile 5 (NR) vs. Quintile 1 (NR)		Categorical	OR	3.02 (1.39, 6.58)	8	2, 3, 6, 9, 11, 17, 22, 25, 27, 36, 39, 66, 69
65	Abbasalizad Farhangi et al. 2018	Cross-sectional	Iran	35–80	F	120	FFQ	120	–	28	HbA1C (%)	Morbidity	Quartile 4) − 29.83 to ≤− 15.05) vs. Quartile 1(− 0.19 to ≤ 7.01(		Categorical	β-coefficient	0.88 (0.59, 1. 31)	6	2, 6, 9, 17, 25, 67
											TC (mg/dl)						0.67 (0.34, 1.37)		
											TG (mg/dl)						1.08 (0.94, 1.25)		
											LDL-C (mg/dl)						1.46 (0.72, 2.97)		
											HDL-C (mg/dl)						1.42 (0.70, 2.88)		
											Lipoprotein (a) (mg/dl)						0.98 (0.96, 1.00)		
					M	332		332			HbA1C (%)						0.89 (0.71–1.12)		
											TC (mg/dl)						1.02 (0.99–1.04)		
											TG (mg/dl)						0.99 (0.98–0.99)		
											LDL-C (mg/dl)						1.001 (0.98–1.02)		
											HDL-C (mg/dl)						− 0.95 (0.91–1.00)		
											Lipoprotein (a) (mg/dl)						1.01 (0.99–1.02)		
66	Luglio Muhammad et al. 2018	Cross-sectional	Indonesia	19–56	F/M	503	FFQ	503	–	30	BMI (kg/m2)	Morbidity	–		Continues (per one unit)	β-coefficient (SE)	− 0.08 (0.036)	6	1, 2, 3, 9, 17
											Body weight (kg)						− 0.03 (0.09)		
											Body fat (%)						− 0.04 (0.04)		
											WC (cm)						− 0.04 (0.09)		
											HC (cm)						− 0.04 (0.07)		
											SBP (mmHg)						0.03 (0.16)		
											DBP (mmHg)						0.04 (0.10)		
											TG (mmol/L)						0.04 (0.006)		
											HDL-C (mmol/L)						− 0.04 (0.004)		
67	Alam et al. 2018	Cross-sectional	Pakistan	54–95	M	651	24-dietary recall	651	–	NR	Body weight (kg)	Morbidity	-		Categorical	Tertile 3 (Mean ± SD)	69.05 ± 10.2	8	-
											BMI (kg/m^2^)						24 ± 1.8		
											WC (cm)						85.5 ± 7.4		
											WHR						0.99 ± 0.11		
68	Kim et al. 2018	Cross-sectional	Korea	19–65	F	5609	24-h dietary recall	1044	–	23	Abdominal obesity	Morbidity	Quartile 4 (≥ 1.28) vs. Quartile 1 (< -0.85)		Categorical	OR	1.35 (0.94, 1.94)	8	1, 2, 3, 7, 9, 25, 39
								2092			Low HDL-C						0.85 (0.71, 1.04)		
								1060			Hyper- triglyceridemia						1.07 (0.84, 1.38)		
								1335			HTN						1.10 (0.87, 1.38)		
								1292			Hyperglycemia						0.95 (0.77, 1.18)		
								966			MetS						1.22 (0.91, 1.64)		
					M	3682		1010			Abdominal obesity		Q4 (≥ 1.89) vs. Q1 (< − 0.16)				1.07 (0.72, 1.61)		
								902			Low HDL-C						0.93 (0.71, 1.21)		
								1489			Hyper- triglyceridemia						1.22 (0.97, 1.53)		
								1429			HTN						1.14 (0.88, 1.46)		
								1384			Hyperglycemia						1.30 (1.02, 1.65)		
								1043			MetS						1.40 (1.06, 1.85)		
69	Correa-Rodriguez et al. 2018	Cross-sectional	Spain	9–17	F/M	428 F (242) M (186)	24-h dietary recall	428	–	28	BMI z-score	Morbidity	–		Continues (per one unit)	β-coefficient	0.084 (− 0.015, 0.116)	7	1, 9, 17
											WC (cm)						0.100 (− 0.060, 1.296)		
											WHtR						0.128 (0.001, 0.016)		
											WHR						0.004 (− 0.004, 0.004)		
											FM (kg)						0.069 (− 0.182, 0.859)		
											PFM (%)						0.050 (− 0.318, 0.885)		
											FFM (kg)						0.045 (− 0.240, 0.735)		
											SBP (msmHg)						0.010 (− 0.933, 1.114)		
											DBP (mmHg)						− 0.032 (− 0.960, 0.540)		
70	Mazidi et al. 2018	Cross-sectional	USA	≥ 18	F/M	17,689 F (9,082) M (8,607)	24-h dietary recall	NR	–	18	MetS	Morbidity	Q4 (1.62 to 4.24) vs. Q1 (− 5.66 to -1.04)		Categorical	OR	1.23 (1.07, 1.41)	8	1, 2, 7, 9, 17, 18, 25, 29
											Obesity						1.28 (1.17, 1.52)		
											HTN						1.21 (1.02, 1.43)		
71	Mirmajidi et al .2018	Cross-sectional	Iran	18–60	F/M	150 F (74) M (76)	FFQ	150		34	BMI (kg/m^2^)	Morbidity	-		Continuous (per one unit)	β-coefficient	0.351 (0.258, 1.247)	6	1, 3, 9, 17
											FBS (mg/dl)						0.402 (0.826, 3.040)		
											Insulin (mg/dl)						0.166 (− 0.425, 2.217)		
											HOMA-IR (mg/dl)						0.214 (− 0.038, 0.590)		
											HOMA-B (mg/dl)						0.112 (− 0.160, 0.433)		
											QUICKI						− 0.239 (− 0.009, 0.000)		
											Chemerin (ng/mL)						0.317 (59.09, 331.45)		
											Omentin (ng/ml)						− 0.192 (29.272, 3.405)		
											LBP (mg/ml)						0.223 (0.469, 3.146)		
72	Moe San et al. 2018	Cross-sectional	Myanmar	25–60	F	244	24-h dietary recall and Semi-quantitative FFQ	116	–	31	High BMI	Morbidity	Higher DII (> 1.07) vs. lower DII (< 1.07)		Categorical	OR	1.40 (0.80, 2.30)	6	2, 9, 29, 68
								91			Abdominal obesity						1.40 (0.80, 2.40)		
								196			Body fat mass						1.10 (0.50, 2.10)		
73	Nikniaz Et al. 2018	Cross-sectional	Iran	18–64	F/M	606 F (324) M (282)	FFQ	NR	–	30	Abdominal obesity	Morbidity	Quartile 4 (NR) vs. Quartile 1 (NR)		Categorical	OR	0.86 (0.39, 1.91)	7	2, 3, 7, 9, 17
											Low HDL-C						0.83 (0.44, 1.55)		
											Hyper- triglyceridemia						1.31 (0.66, 2.58)		
											HTN						1.18 (0.47, 2.96)		
											Hyperglycemia						2.56 (1.01, 7.05)		
											MetS						2.26 (1.03, 4.92)		
75	Park et al. 2018	Cross-sectional	Korea	≥ 50	F	1344	24-h dietary recall	334	-	42	Osteopenic obesity	Morbidity	Higher DII (> − 0.07) vs. lower DII (≤ − 0.07)		Categorical	OR	2.757 (1.398, 5.438)	8	2, 7, 9, 25, 37, 53
								110			Sarcopenic obesity						1.968 (0.951, 4.073)		
								445			Steosarcopenic obesity						2.186 (1.182, 4.044)		
74	Shivappa et al. 2018	Case–control	Iran	18–40	F	388	FFQ	122	–	32	GDM	Morbidity	Tertile 3 (> − 0.38) vs. tertile 1 (≤ − 1.32)		Categorical	OR	2.10 (1.02, 4.34)	7	1, 2, 3, 6, 7, 9, 27
													-		Continuous (per one unit)		1.20 (0.94, 1.54)		
45	Winkvist et al. 2018	Cohort	Sweden	30–60	F	8345	FFQ	NR	10	30	BMI (kg/m^2^)	Morbidity	-		Continuous (per one percent)	β-coefficient ± SE	0.000 ± 0.001	8	3, 7, 9, 25, 63
											TG (mmol/l)						0.000 ± 0.000		
											TC (mmol/l)						0.000 ± 0.000		
											SBP (mmHg)						− 0.006 ± 0.003		
					M	7641					BMI (kg/m^2^)						0.000 ± 0.001		
											TG (mmol/l)						0.000 ± 0.000		
											TC (mmol/l)						0.000 ± 0.000		
											SBP (mmHg)						− 0.001 ± 0.004		
76	Ren et al. 2018^a^	Cross- sectional	China	18–75	F/M	1712 F (1130) M (582)	24-h dietary recall	NR	–	21	Abdominal obesity	Morbidity	Tertile 3 (1.12 to 3.49) vs. tertile 1 − 3.50 to 0.04)		Categorical	OR	0.86 (0.59–1.24)	8	2, 7, 9, 17, 25
													-		Continues (per one unit)		0.93 (0.81–1.06)		
											Low HDL-C		Tertile 3 (1.12 to 3.49) vs. tertile 1 − 3.50 to 0.04)		Categorical		1.17 (0.88–1.56)		
													-		Continues (per one unit)		1.02 (0.92–1.12)		
											Hyper- triglyceridemia		Tertile 3 (1.12 to 3.49) vs. tertile 1 − 3.50 to 0.04)		Categorical		1.03 (0.78–1.37)		
													-		Continues (per one unit)		0.99 (0.90–1.09)		
											HTN		Tertile 3 (1.12 to 3.49) vs. tertile 1 -3.50 to 0.04)		Categorical		1.40 (1.03–1.89)		
													-		Continues (per one unit)		1.06 (0.96–1.18)		
											Hyperglycemia		Tertile 3 (1.12 to 3.49) vs. tertile 1 − 3.50 to 0.04)		Categorical		0.85 (0.64–1.14)		
													-		Continues (per one unit)		0.91 (0.82–1.00)		
											MetS		Tertile 3 (1.12 to 3.49) vs. tertile 1 − 3.50 to 0.04)		Categorical		1.02 (0.75–1.40)		
													-		Continues (per one unit)		0.93 (0.83–1.04)		

1—total energy intake, 2—body mass index, 3—physical activity, 4—systolic blood pressure, 5—total cholesterol, 6—diabetes, 7—smoking, 8—postsecondary academic education, 9—age, 10—energy expended in physical activity, 11—socioeconomic status, 12—use of low-dose aspirin, 13—use of antihypertensive medication, 14—use of statins, 15—prevalent atherosclerotic vascular disease, 16—treatment code, 17—sex, 18—race, 19—HbA1c, 20—overweight/obesity, 21—waist to height ratio, 22—hypertension, 23—dyslipidemia, 24—family history of premature cardiovascular disease, 25—educational level, 26—stratified by intervention group and center, 27—supplementation, 28—number of 24-h records, 29—marital status, 30—treatment allocation group (placebo or active), 31—diastolic blood pressure, 32—waist circumference, 33—previous history of other cardiovascular diseases, 34—following a special diet, 35—hours spent sitting down, 36—hours spent watching television, 37—hormone replacement therapy use, 38—prevalent cancer (yes/no), 39—alcohol intake, 40—survey number, 41—place of residence, 42—ratio of total cholesterol and high density lipoprotein cholesterol, 43—poverty index, 44—coffee consumption, 45—menopausal status, 46—family member, 47—occupational grade, 48—use of lipid-lowering drugs, 49—high density lipoprotein cholesterol, 50—longstanding illness, 51—country of birth, 52—socio-economic indexes for areas quintile, 53—income, 54—glucose lowering medication, 55—crowding index, 56—number of available dietary records, 57—snacking between meals, 58—parental history of obesity, 59—depression (previous or incident), 60—analgesic use, 61—triglyceride, 62—low density lipoprotein cholesterol, 63—year of study participation, 64—years of police work, 65—history of chronic diseases, 66—medication use, 67—myocardial infarctioyn, 68—use of contraceptives, 69—tobacco use

*CMRFs* cardio-metabolic risk factors, *DII* dietary inflammatory index, *F* female, *M* male, *FFQ* food frequency questionnaire, *HDL-C* high density lipoprotein-cholesterol, *LDL-C* low density lipoprotein-cholesterol, *VLDL* very low density lipoprotein, *LBP* lipopolysaccharide-binding protein, *TC* total cholesterol, *TG* triglyceride, *TC* total cholesterol, *HTN* hypertension, *SBP* systolic blood pressure, *DBP* diastolic blood pressure, *MetS* metabolic syndrome, *OR* odds ratio, *HbA1c* glycated hemoglobin, *FBS* fasting blood sugar, *HOMA-IR* homeostasis model assessment of insulin resistance, *HOMA-B* homeostatic model assessment of β-cell function, *QUICKI* quantitative insulin-sensitivity check index, *IFG* impaired fasting glucose, *IGT* impaired glucose tolerance, *OGTT* oral glucose tolerance test, *GDM* gestational diabetes mellitus, *BMI* body mass index, *WC* waist circumference, *HC* hip circumference, *WHR* waist to hip circumference, *WHtR* waist to height ratio, *FM* fat mass, *FFM* fat free mass, *PFM* percentage fat mass, *VFR* visceral fat ratio, *SS* *+* *Tr* subscapular + triceps skinfold thickness, *LBM* lean body mass, *DXA* Dual energy X-ray absorptiometry, *FMD* flow-mediated vasodilation, *PWV* pulse wave velocity, *MAP* mean arterial pressure, *MUO* metabolically unhealthy obese, *NR* Not reported

### Results of qualitative synthesis

#### Association between the DII score with risk of CMDs and mortality

The positive association between the DII score (as a continuous variable) and risk of CMDs and mortality was observed in three [16, 19, 57] and six [29, 30, 54, 56, 78, 79] studies, respectively. Moreover, three records did not indicate the significant association between the DII score and risk of CMDs [31, 32, 52]. In addition, two studies failed to find any significant association between the DII score and risk of CMDs mortality [31, 55].

The DII score (as a categorical variable) was associated significantly with the risk of CMDs in six studies [15–17, 19, 53, 57] and seven reports showed the positive association between the index and risk of CMDs mortality [29, 51, 54, 56, 77–79]. Furthermore, three studies did not demonstrate any significant association between the DII score and risk of CMDs [31, 32, 52]. Moreover, three studies reported no significant association between this index and risk of CMDs mortality [30, 31, 55]. In one study, a significant association was observed between the DII score and risk of CMDs mortality only in normal and pre-diabetic participants [51].

#### Association between DII with CMRFs

Totally, 39 studies (28 cross-sectional study [19, 20, 25, 26, 28, 42, 43, 46, 47, 49, 50, 58, 60–73, 75, 76], nine cohort study [17, 22–24, 27, 44, 45, 48, 59] and two case–control studies [21, 74]) had assessed CMRFs as an outcome [17, 19–28, 42–50, 58–76].The lowest and highest reported ORs were observed for the association between the DII score and abdominal obesity [OR: 0.58 (95% CI 0.16, 2.05)] [58] and morbidity of pre-diabetes [OR: 18.88 (95% CI 7.02, 50.82)] [21], respectively. Nine studies reported no association between the DII score and abdominal obesity [20, 25, 26, 28, 58, 68, 72, 73, 76]. Two reports illustrated a significant association between the DII score and low level of high-density lipoprotein cholesterol (HDL-C) [26, 28], whereas six studies failed to find this association [20, 25, 58, 68, 73, 76]. With respect to hypertriglyceridemia, eight studies reported no association between this score and hypertriglyceridemia [20, 25, 26, 28, 58, 68, 73, 76]. The DII score was associated with HTN in five studies [17, 19, 24, 70, 76] and eight studies did not show any significant association [20, 25–28, 58, 68, 73]. Moreover, one study reported no association between the DII score and gestational HTN [27]. Six studies reported no association between the DII score and hyperglycemia [25–28, 58, 76], whereas two studies revealed this association [20, 73]. Another study indicated a positive association between this score and hyperglycemia only in men [68]. Also, four studies reported a positive association between the DII score and MetS [23, 62, 70, 73]; six studies reported no association in this regard [20, 25, 26, 28, 61, 76]. Moreover, one study demonstrated a significant association between this score and MetS only in men [68]. In terms of body mass index (BMI), four studies showed no association between the DII score and BMI [42, 45, 63, 66], whereas two studies indicated a significant association [49, 71]. Another report found a significant association between the DII score and BMI only in women [47]. One cohort study showed a significant association between the DII score and BMI z-score in boys [44]; another study failed to find any association between the DII score and BMI z-score [69]. Moreover, another study indicated this association in all students [60]. A significant association between the DII score and low density lipoprotein cholesterol (LDL-C) levels was observed in two studies [21, 65] and three studies failed to find any association [42, 44, 48]. The DII score was associated with total cholesterol (TC) levels only in one study [65], whereas three studies did not show this association [42, 45, 48]; another study reported no association between the DII score and hypercholesterolemia [17].

### Quality assessment

According to NOS, 49 studies had high quality (NOS ≥ 7) [15–17, 21–32, 42–64, 67–70, 73–79], and four studies obtained 6 stars [65, 66, 71, 72]. Only, two reports achieved NOS = 5 [19, 20].

### Results of meta-analysis

#### DII score and risk of CMDs and mortality

**Fig. 2:**
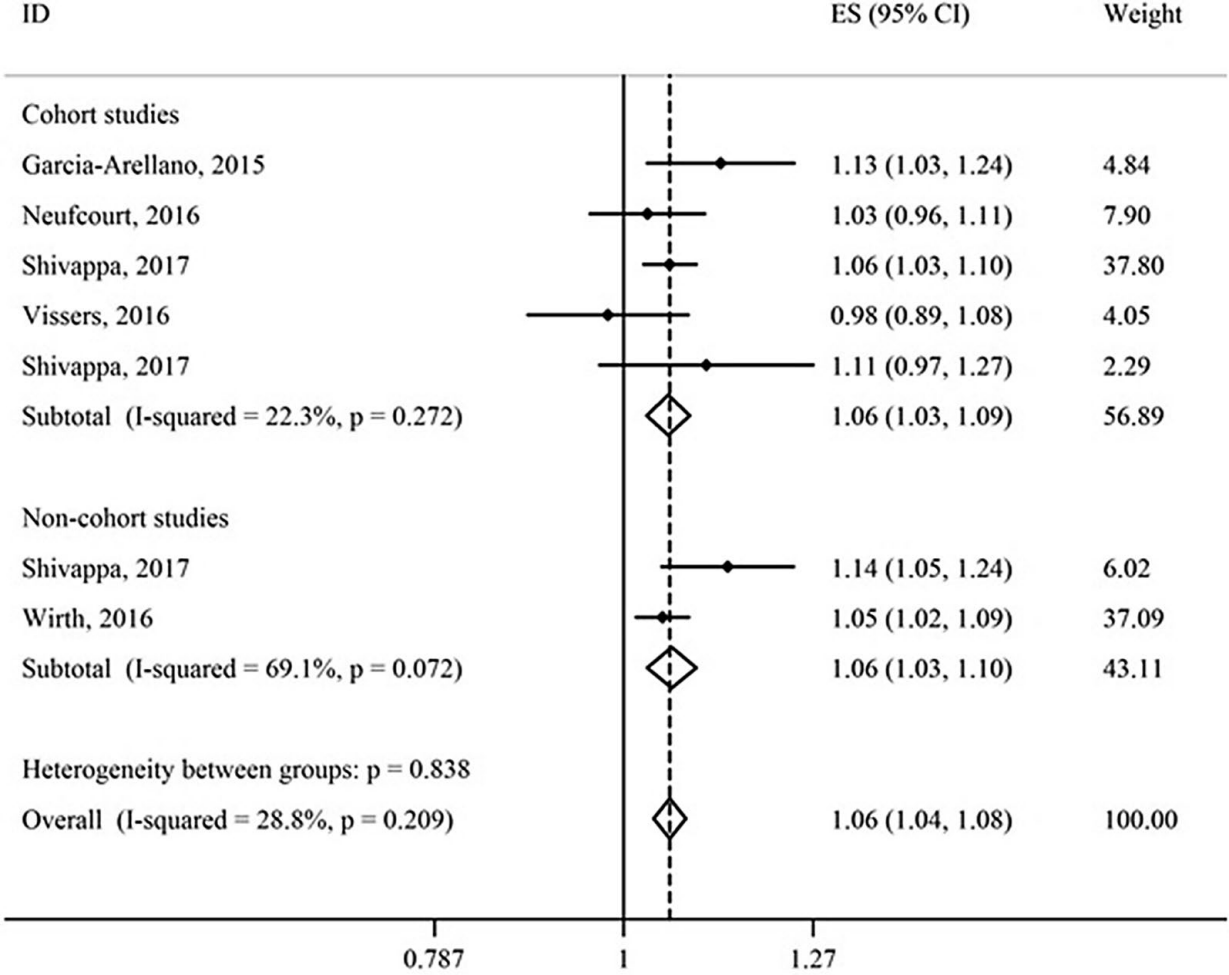
Association of dietary inflammatory index (DII) (as a continuous variable) and risk of cardiometabolic diseases

**Fig. 3:**
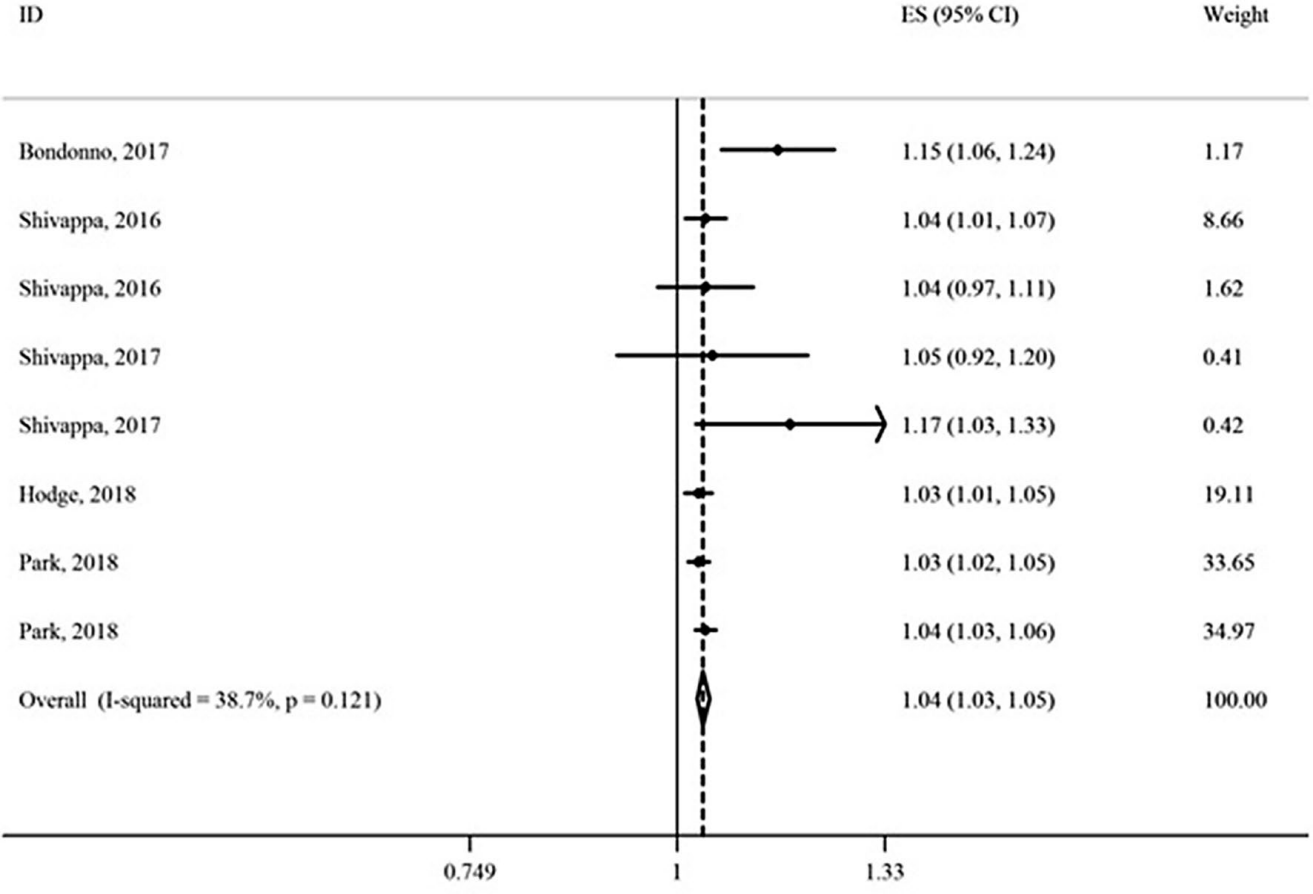
Association of dietary inflammatory index (DII) (as a continuous variable) and risk of cardiometabolic diseases mortality

Thirteen studies that investigated the association between the DII score (as a continuous variable) and risk of CMDs and mortality were included in this meta-analysis [16, 19, 29–32, 52, 54–57, 78, 79] (Figs. 2 and 3). Subgroup analysis was performed according to the type of outcome (morbidity/mortality) and study design (cohort/non-cohort) (Table 4). Results of fixed effect meta-analysis showed that per one-unit increment in the DII score the risk of CMDs mortality increased significantly by 4% (HR = 1.04; 95% CI 1.03, 1.05). Also, a significant association was observed between the continuous DII and risk of CMDs in cohort (HR = 1.06; 95% CI 1.03, 1.09) and non-cohort studies (HR = 1.06; 95% CI 1.03, 1.10).

**Table 4:** Meta-analysis of association between continuous and categorical dietary inflammatory index (DII) and risk of cardiometabolic diseases and mortality according to type of study

Type of the DII measurement	Type of outcome	Type of study	Number of studies	Sample size	Number of events	Type of effect size measures	Test of association		Test of heterogeneity		
							Effect size measure	95% CI	Model	I^2^%	p-value
Continuous (per one unit increment)	Mortality	Cohort	8	239,156	27,403	HR^a^	1.04	1.03–1.05	Fixed	38.7	0.12
	Morbidity	Cohort	4	23,183	1117	HR	1.06	1.03–1.09	Fixed	22.3	0.27
		Non-cohort^b^	2	17,055	2494	OR^a^^,^^b^	1.06	1.03–1.10	Random	69.1	0.07
Categorical (highest DII/ lowest DII)	Mortality	Cohort	10	291,968	30,813	HR	1.29	1.18–1.41	Random	65.9	< 0.001
	Morbidity	Cohort	6	43,340	1310	HR	1.35	1.13–1.61	Fixed	37.0	0.16
		Non-cohort ^b^	3	23,999	3883	OR^b^	1.36	1.18–1.57	Fixed	0.0	0.67

*OR* odds ratio, *HR* hazard ratio, *CI* confidence interval

^a^HR, Hazard ratio; OR, Odds ratio; Q test, Cochran test

^b^Case–control or cross-sectional study

**Fig. 4:**
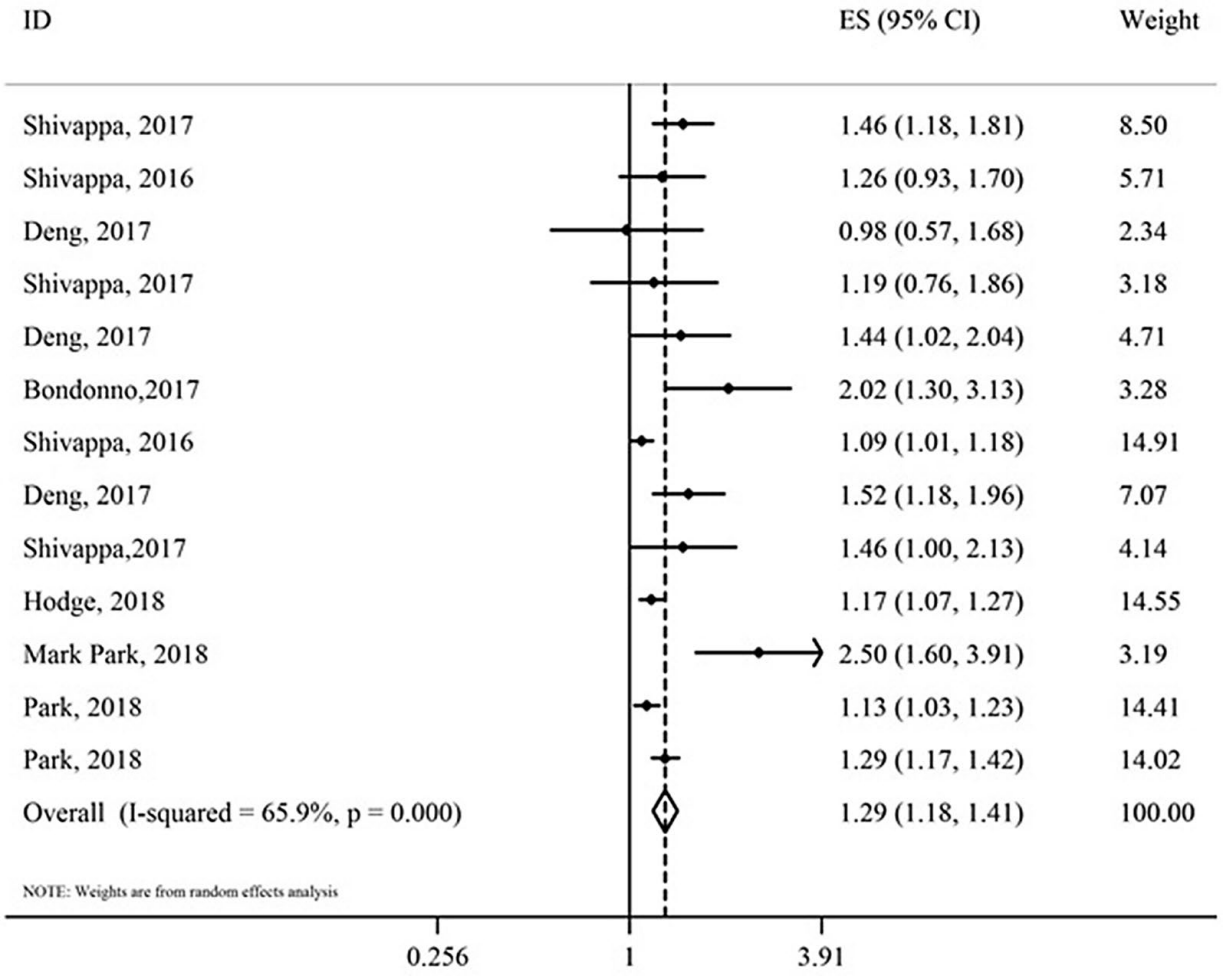
Association of dietary inflammatory index (DII) (as a categorical variable) with risk of cardiometabolic diseases mortality

We also assessed the association between the categorical DII score and risk of CMDs and mortality using 18 observational studies [15–17, 19, 29–32, 51–57, 77–79]. Meta-analysis of cohort studies showed that the most pro-inflammatory diet category (the highest DII score group) compared to the most anti-inflammatory diet category (the lowest DII score group), increases the risk of CMDs mortality by 29% (HR = 1.29; 95% CI 1.18, 1.41) (Fig. 4).

**Fig. 5:**
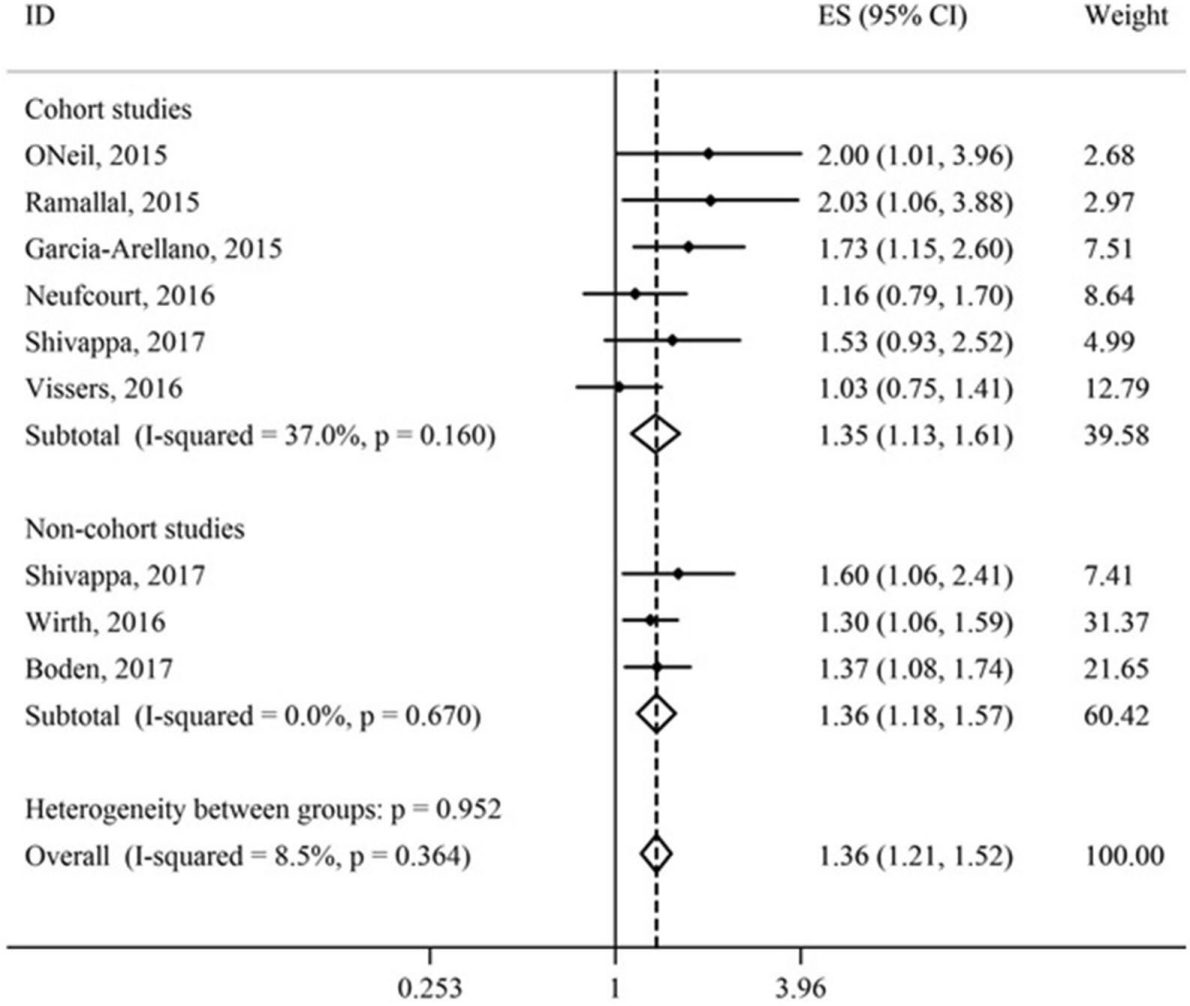
Association of dietary inflammatory index (DII) (as a categorical variable) with risk of cardiometabolic diseases

Also, the association between the DII and risk of CMDs was statistically significant in cohort (HR = 1.35; 95% CI 1.13, 1.61) and non-cohort studies (HR = 1.36; 95% CI 1.18, 1.57) (Fig. 5).

#### DII score and CMRFs

**Fig. 6:**
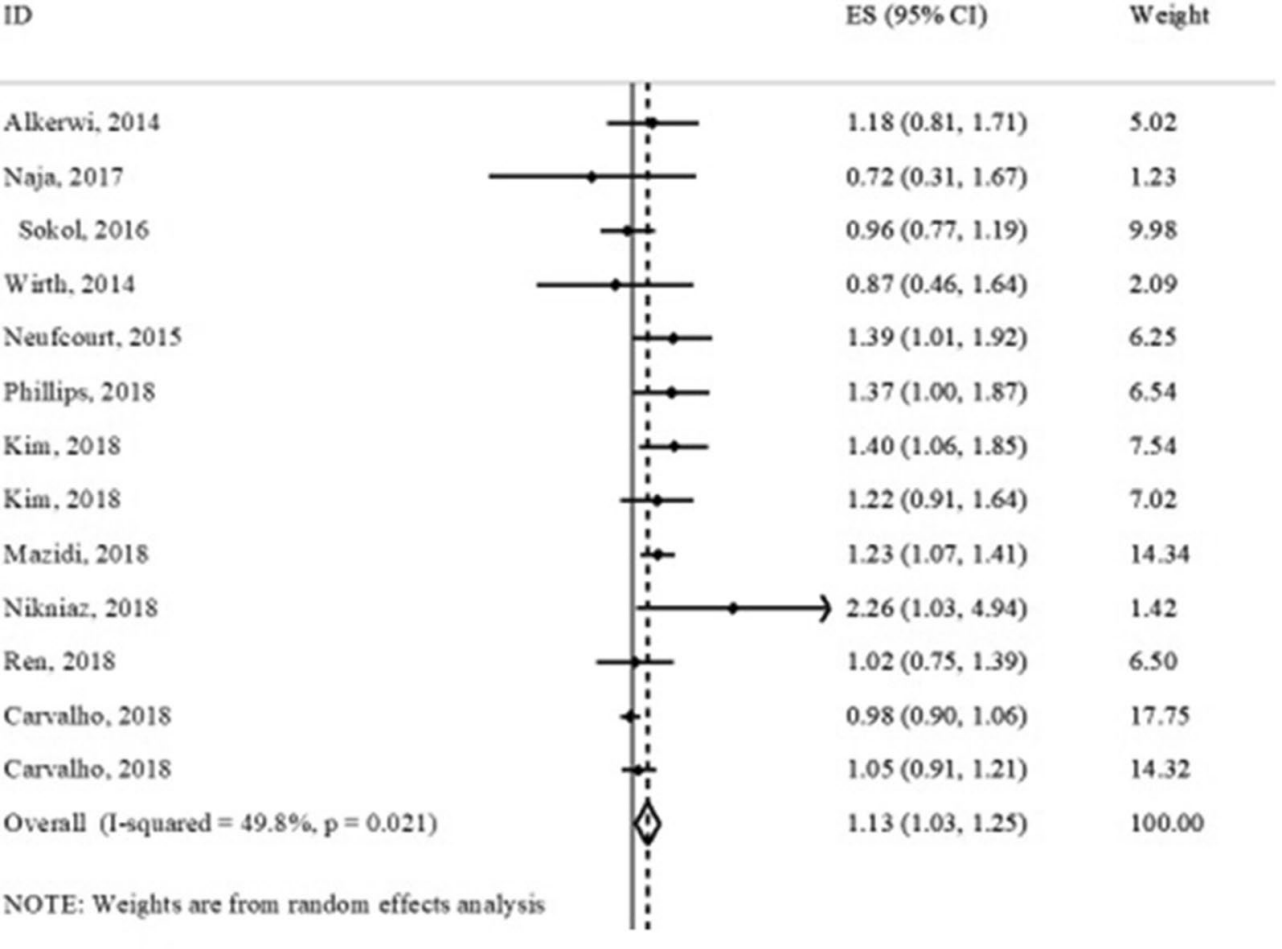
Association between dietary inflammatory index (DII) and metabolic syndrome

Of 39 publications, 16 studies had assessed the association between the DII score and MetS or at least one of its components and had reported measure of association (OR) included in the meta-analysis [17, 19, 20, 23–26, 28, 58, 61, 62, 68, 70, 72, 73, 76] (Table 5). Results of meta-analysis indicated a significant association between the DII score and MetS (OR: 1.13; 95% CI 1.03–1.25) (Fig. 6), hyperglycemia (OR: 1.21; 95% CI 1.01–1.44) and HTN (OR: 1.17; 95% CI 1.10–1.25). We failed to find any significant association between the DII score and other components of MetS (abdominal obesity, low HDL-C and hyper-triglyceridemia).

**Table 5:** Meta-analysis of association between dietary inflammatory index (DII) (as a categorical index) and cardiometabolic risk factors

Outcome variable	Number of studies	Sample size	Number of events	Test of association		Test of heterogeneity		
				OR^a,d^	95% CI	Model	I^2^%	p-value
Abdominal obesity	9	18,121	4655^b^	1.00	0.88–1.12	Fixed	3.5	0.40
Low HDL-C	8	17,874	4148^b^	0.94	0.78–1.14	Random	58.1	0.01
Hyper- triglyceridemia	8	17,874	3954^b^	1.09	0.98–1.22	Fixed	0.0	0.73
HTN	12	77,194	13,496^c^	1.17	1.10–1.25	Fixed	36.4	0.12
Hyperglycemia	8	17,876	4651^b^	1.21	1.01–1.44	Random	54.0	0.02
MetS	11	42,978	4524^b^	1.13	1.03–1.25	Random	54.8	0.02

*HDL-C* high density lipoprotein-cholesterol, *HTN* hypertension, *MetS* metabolic syndrome, *OR* odds ratio, *CI* confidence interval

^*^HR, Hazard ratio; OR, Odds ratio; Q test, Cochran test

^a^Cohort or cross-sectional study

^b^Participants with abdominal obesity, low-HDL-C, hyper-triglyceridemia, hyperglycemia and MetS had not been stated in three studies

^c^Participants with HTN had not been stated in five studies

^d^ The odds ratio is for the highest pro-inflammatory diet (the highest DII) versus the highest anti-inflammatory diet (the lowest DII)

^e^Case–control or cross-sectional study

### Results of dose–response meta-analysis

In the terms of risk of CMDs mortality in relation to the DII score, nine cohort studies [29, 31, 51, 54–56, 77–79] were included in dose–response analysis. A significant non-linear positive association was found between the DII score and CMDs mortality (P_nonlinearity_ < 0.001). Unlike the overall association, the DII score was inversely associated with CMDs mortality from score of − 5 to − 2 (P_nonlinearity_ = 0.01). However, the risk was significantly increased when increasing the score of DII from − 2 to 1.5 (P_nonlinearity_ < 0.001). The slope was slightly flattening from DII score of 1.5 to upper levels (Additional file 3: Figure S1).

Six studies (four cohorts [16, 17, 31, 52], one case–control [57] and one cross-sectional study [19]) were included in dose–response analysis assessing the association between the DII score and risk of CMDs (Additional file 4: Figure S2). No significant non-linear association was found in this regard (p-value = 0.1). Such non-significant association was also seen after considering only cohort studies and excluding case–control and cross-sectional studies (p-value = 0.2) (Additional file 5: Figure S3).

### Publication bias

No publication bias was observed between studies of MetS according to Egger test results (p-value = 0.323). Moreover, the results of Egger test for studies evaluated the association between the continuous DII score and risk of CMDs and mortality showed that there was no evidence of publication bias between studies (p-value = 0.114, p-value = 0.745, respectively) (Additional file 6: Figures S4 and Additional file 7: Figure S5). When we considered studies with the categorical DII score, the publication bias was observed in our analysis (P_Egger_ = 0.001 for risk of CMDs and P_Egger_ = 0.04 for risk of CMDs mortaliy) (Additional file 8: Figure S6 and Additional file 9: Figure S7).

### Sensitivity analysis

Sensitivity analysis showed that removing any of the studies or a group of studies could not significantly change the effect of DII score (as a continuous or categorical variable) on risk of CMDs and mortality. In terms of MetS and its components, the results of sensitivity analysis demonstrated that neither an individual study nor group of studies had a remarkable effect on our results.

## Discussion

The present meta-analysis showed evidences of the association between increasing the inflammatory potential of diet and risk of CMDs and mortality. Also, individuals with the highest pro-inflammatory diet had 13%, 21%, and 17% higher risk for MetS, hyperglycemia and HTN than those with the lowest pro-inflammatory diet.

Subgroup analysis showed that the association of DII (as continuous and categorical variable) with risk of CMDs did not change appreciably in the cohort and non-cohort studies. One important issue in studies on the association of the dietary indices and chronic diseases is the sample size. We can find more precise results using larger sample sizes. Similar findings in the cohort and non-cohort studies can be probably explained by the larger sample size of non-cohort studies.

In the current study, there was a significant association between the DII score and risk of CMDs and mortality. There are some theories that explain the relationship between the DII score and risk of CVDs. Findings of studies showed that higher consumption of pro-inflammatory foods such as red and processed meat, sugar, and refined grains increases level of IL-6, TNF-a, and hs-CRP [12]. Higher levels of these inflammatory biomarkers is the main etiologic factor in CMDs development [80–84]. Since the DII score was calculated using dietary factors (nutrients and specific food items) which show the diet-associated inflammation [14], it was anticipated to observe an association between the DII score and risk of CMDs.

A population-based study including 1,363 men aged 18 years and older (the Geelong osteoporosis study) showed that the adjusted OR (95% CI) for CVDs was 2 (1.01–3.96) for those with pro-inflammatory diet compared with anti-inflammatory diet [53]. The PREDIMED study investigated 7,216 men aged 55–80 years and women aged 60–80 years at high risk of CVDs. A total of 277 CVDs events were considered. The adjusted hazard ratio (95% CI) for CVDs was 1.73 (1.15–2.60) for participants with pro-inflammatory diet. A stronger relationship was showed when cases occurring during the first year of follow-up were excluded from the analysis [16]. Moreover, the in SU.VI.MAX study included 7743 women aged 35–60 years and men aged 45–60 years with 11.4 years follow-up, no statistically significant association was found between the DII score and the composite CVDs outcome. However, a significant relationship was shown for MI when the highest quartile was compared with the lowest quartile of the DII score [52]. Moreover, another cross-sectional study carried out on Sweden men and women aged 30–73 years showed a positive association between the DII score and risk of CMDs [15]. A cohort study on a large sample size of Sweden women indicated that there is not association between the DII score and risk of CVDs mortality [55]. This finding may related to the low number of used dietary factors in DII calculation. In another cohort study on diabetic patients, results showed that there is not any association between the DII score and risk of CVDs mortality that it is not in line our study. This finding can be related to the low sample size and dietary factors used for DII calculation [51].

This meta-analysis of 16 studies examining the association between the DII score and MetS or at least one of its components [17, 19, 20, 23–26, 28, 58, 61, 62, 68, 70, 72, 73, 76], showed a significant association between the DII score and MetS, hyperglycemia and HTN. Several population-based studies carried out in France, Ireland, USA and Iran demonstrated the significant association between the DII score and MetS [23, 62, 70, 73]. However, other studies failed to find this association [20, 25, 26, 28, 61, 76]. Ramallal et al. [17] in a cohort study on 18,794 Spanish men and women showed the higher DII score is associated with greater incidence of HTN. Also, other studies indicated this significant association [19, 24, 70, 76]. In the regard of hyperglycemia, studies carried out in USA and Iran indicated a positive association between the DII score and hyperglycemia [20, 73]. However, some studies did not demonstrate this association [25, 26, 28, 58].

The meta-analysis of 14 studies revealed that subjects in the highest versus the lowest DII score category showed 36% increased risk of CVDs incidence and mortality [33]. Another meta-analysis found that participants with higher DII score had a higher risk of cardiovascular and cancer mortality [30]. The strengths of our study against the other two meta-analyses include the evaluation of the association between the DII score and CMRFs and the dose–response association between the DII score and risk of CMDs and mortality. In addition, we assessed the risk of CMDs separately in all cohort and non-cohort studies.

The current study had several limitations. Absence of a specific cut-off point for the association of the DII score and occurrence of morbidity or mortality of CMDs is the first limitation. Most of studies included in the MetS and its components analyses had a cross-sectional design, so the limitations of this type of study should be considered and the results should be interpreted with cautious. Other limitations include different numbers of dietary factors used in the DII score calculation and applying different adjustment models in the analyses. Evidence of publication bias, the other limitation, was observed when the DII score was considered as a categorical variable in the analyses.

## Conclusion

The current meta-analysis study showed a positive association between the DII score and risk of CMDs and mortality. Also, we find a significant association between adherence to pro-inflammatory diet and MetS, hyperglycemia, and HTN. More studies with prospective designs and in different societies are needed to confirm the findings.

## Supplementary information

**Additional file 1: Appendix S1.** PRISMA 2009 Checklist.

**Additional file 2: Table S1.** Search strategy in PubMed.

**Additional file 3: Figure S1.** Dose–response association between the DII and risk of cardiometabolic diseases mortality.

**Additional file 4: Figure S2.** Dose–response association between the DII and risk of cardiometabolic diseases.

**Additional file 5: Figure S3.** Dose–response association between the DII and risk of cardiometabolic diseases in cohort studies.

**Additional file 6: Figure S4.** Funnel plot of dietary inflammatory index (DII) (as a continuous variable) with risk of cardiometabolic diseases.

**Additional file 7: Figure S5.** Funnel plot of dietary inflammatory index (DII) (as a continuous variable) with risk of cardiometabolic diseases mortality.

**Additional file 8: Figure S6.** Funnel plot of dietary inflammatory index (DII) (as a categorical variable) with risk of cardiometabolic diseases.

**Additional file 9: Figure S7.** Funnel plot of dietary inflammatory index (DII) (as a categorical variable) with risk of cardiometabolic diseases mortality.

## Data Availability

All data generated or analyzed in this study are included in this published article [and its additional information files].

## References

[1] Libby P (2007). Inflammatory mechanisms: the molecular basis of inflammation and disease. Nut Rev.

[2] Hansson GK (2005). Inflammation, atherosclerosis, and coronary artery disease. N Engl J Med.

[3] Galassetti P (2012). Inflammation and oxidative stress in obesity, metabolic syndrome, and diabetes. J Diabetes Res..

[4] Keibel A, Singh V, Sharma MC (2009). Inflammation, microenvironment, and the immune system in cancer progression. Curr Pharm Des.

[5] Gregor MF, Hotamisligil GS (2011). Inflammatory mechanisms in obesity. Annu Rev Immunol.

[6] Musani SK, Vasan RS, Bidulescu A, Liu J, Xanthakis V, Sims M (2013). Aldosterone, C-reactive protein, and plasma B-type natriuretic peptide are associated with the development of metabolic syndrome and longitudinal changes in metabolic syndrome components: findings from the Jackson Heart Study. Diabetes Care.

[7] Esmaillzadeh A, Kimiagar M, Mehrabi Y, Azadbakht L, Hu FB, Willett WC (2007). Dietary patterns and markers of systemic inflammation among Iranian women. J Nutr.

[8] Fung TT, Rexrode KM, Mantzoros CS, Manson JE, Willett WC, Hu FB (2009). Mediterranean diet and incidence of and mortality from coronary heart disease and stroke in women. Circulation.

[9] Martínez-González MA, García-López M, Bes-Rastrollo M, Toledo E, Martínez-Lapiscina EH, Delgado-Rodriguez M (2011). Mediterranean diet and the incidence of cardiovascular disease: a Spanish cohort. Nutr Metab Cardiovasc Dis.

[10] Trichopoulou A, Martínez-González MA, Tong TY, Forouhi NG, Khandelwal S, Prabhakaran D (2014). Definitions and potential health benefits of the Mediterranean diet: views from experts around the world. BMC Med.

[11] Martinez-Gonzalez MA, Bes-Rastrollo M (2014). Dietary patterns, Mediterranean diet, and cardiovascular disease. Curr Opin Lipidol.

[12] Lopez-Garcia E, Schulze MB, Fung TT, Meigs JB, Rifai N, Manson JE (2004). Major dietary patterns are related to plasma concentrations of markers of inflammation and endothelial dysfunction. Am J Clin Nutr.

[13] Cavicchia PP, Steck SE, Hurley TG, Hussey JR, Ma Y, Ockene IS (2009). A new dietary inflammatory index predicts interval changes in serum high-sensitivity C-reactive protein. J Nutr.

[14] Shivappa N, Steck SE, Hurley TG, Hussey JR, Hébert JR (2014). Designing and developing a literature-derived, population-based dietary inflammatory index. Public Health Nutr.

[15] Bodén S, Wennberg M, Van Guelpen B, Johansson I, Lindahl B, Andersson J (2017). Dietary inflammatory index and risk of first myocardial infarction; a prospective population-based study. Nutr J.

[16] Garcia-Arellano A, Ramallal R, Ruiz-Canela M, Salas-Salvadó J, Corella D, Shivappa N (2015). Dietary inflammatory index and incidence of cardiovascular disease in the PREDIMED study. Nutrients.

[17] Ramallal R, Toledo E, Martínez-González MA, Hernández-Hernández A, García-Arellano A, Shivappa N (2015). Dietary inflammatory index and incidence of cardiovascular disease in the SUN cohort. PLoS ONE.

[18] Li D, Hao X, Li J, Wu Z, Chen S, Lin J (2018). Dose-response relation between dietary inflammatory index and human cancer risk: evidence from 44 epidemiologic studies involving 1,082,092 participants. Am J Clin Nutr.

[19] Wirth MD, Shivappa N, Hurley TG, Hébert JR (2016). Association between previously diagnosed circulatory conditions and a dietary inflammatory index. Nutr Res.

[20] Wirth M, Burch J, Shivappa N, Violanti JM, Burchfiel CM, Fekedulegn D (2014). Association of a dietary inflammatory index with inflammatory indices and the metabolic syndrome among police officers. J Occup Environ Med.

[21] Vahid F, Shivappa N, Karamati M, Naeini AJ, Hebert JR, Davoodi SH (2016). Association between Dietary Inflammatory Index (DII) and risk of prediabetes: a case-control study. Appl Physiol Nutr Metab.

[22] Ramallal R, Toledo E, Martínez JA, Shivappa N, Hébert JR, Martínez-González MA (2017). Inflammatory potential of diet, weight gain, and incidence of overweight/obesity: the SUN cohort. Obesity.

[23] Neufcourt L, Assmann K, Fezeu L, Touvier M, Graffouillère L, Shivappa N (2015). Prospective association between the dietary inflammatory index and metabolic syndrome: findings from the SU. VI. MAX study. Nutr Metab Cardiovasc Dis.

[24] Alkerwi AA, Shivappa N, Crichton G, Hébert JR (2014). No significant independent relationships with cardiometabolic biomarkers were detected in the Observation of Cardiovascular Risk Factors in Luxembourg study population. Nutr Res..

[25] Naja F, Shivappa N, Nasreddine L, Kharroubi S, Itani L, Hwalla N (2017). Role of inflammation in the association between the western dietary pattern and metabolic syndrome among Lebanese adults. Int J Food Sci Nutr.

[26] Vissers L, Waller M, van der Schouw Y, Hébert J, Shivappa N, Schoenaker D (2017). A pro-inflammatory diet is associated with increased risk of developing hypertension among middle-aged women. Nutr Metab Cardiovasc Dis.

[27] Sen S, Rifas-Shiman SL, Shivappa N, Wirth MD, Hébert JR, Gold DR (2015). Dietary inflammatory potential during pregnancy is associated with lower fetal growth and breastfeeding failure: results from project Viva–3. J Nutr.

[28] Sokol A, Wirth MD, Manczuk M, Shivappa N, Zatonska K, Hurley TG (2016). Association between the dietary inflammatory index, waist-to-hip ratio and metabolic syndrome. Nutr Res.

[29] Bondonno NP, Lewis JR, Blekkenhorst LC, Shivappa N, Woodman RJ, Bondonno CP (2017). Dietary inflammatory index in relation to sub-clinical atherosclerosis and atherosclerotic vascular disease mortality in older women. Br J Nutr.

[30] Vissers LE, Waller MA, van der Schouw YT, Hebert JR, Shivappa N, Schoenaker DA (2016). The relationship between the dietary inflammatory index and risk of total cardiovascular disease, ischemic heart disease and cerebrovascular disease: Findings from an Australian population-based prospective cohort study of women. Atherosclerosis.

[31] Shivappa N, Hebert JR, Kivimaki M, Akbaraly T (2017). Alternative healthy eating index 2010, dietary inflammatory index and risk of mortality: results from the whitehall II cohort study and meta-analysis of previous Dietary Inflammatory Index and mortality studies. Br J Nutr.

[32] Shivappa N, Schneider A, Hébert JR, Koenig W, Peters A, Thorand B (2017). Association between dietary inflammatory index, and cause-specific mortality in the MONICA/KORA Augsburg Cohort Study. Eur J Public Health.

[33] Shivappa N, Godos J, Hébert JR, Wirth MD, Piuri G, Speciani AF (2018). Dietary inflammatory index and cardiovascular risk and mortality—a meta-analysis. Nutrients.

[34] Namazi N, Larijani B, Azadbakht L (2018). Dietary inflammatory index and its association with the risk of cardiovascular diseases, metabolic syndrome, and mortality: a systematic review and meta-analysis. Horm Metab Res..

[35] Zhong X, Guo L, Zhang L, Li Y, He R, Cheng G (2017). Inflammatory potential of diet and risk of cardiovascular disease or mortality: a meta-analysis. Sci Rep.

[36] Wells GA SB, O’Connell D, Peterson J, Welch V, Losos M, Tugwell P. The Newcastle–Ottawa Scale (NOS) for assessing the quality of nonrandomised studies in meta-analyses.Ottawa Hospital Research Institue; 2014. https://www.ohri.ca/programs/clinical_epidemiology/oxford.asp. Accessed June 2016.

[37] Lipsey M, Wilson D (2001). Practical meta-analysis.

[38] Higgins JP, Thompson SG (2002). Quantifying heterogeneity in a meta-analysis. Stat Med.

[39] Cao YXY, Lu T, Gao F, Mo Z (2008). Metan: fixed- and random-effects meta-analysis. Stata J.

[40] Orsini N, Bellocco R, Greenland S (2006). Generalized least squares for trend estimation of summarized dose-response data. Stata J.

[41] Harre FE, Lee KL, Pollock BG (1988). Regression models in clinical studies: determining relationships between predictors and response. J Natl Cancer Inst.

[42] Alkerwi AA, Vernier C, Crichton GE, Sauvageot N, Shivappa N, Hébert JR (2015). Cross-comparison of diet quality indices for predicting chronic disease risk: findings from the Observation of Cardiovascular Risk Factors in Luxembourg (ORISCAV-LUX) study. Br J Nutr..

[43] Moslehi N, Ehsani B, Mirmiran P, Shivappa N, Tohidi M, Hébert JR (2016). Inflammatory properties of diet and glucose-insulin homeostasis in a cohort of iranian adults. Nutrients.

[44] Sen S, Rifas-Shiman S, Shivappa N, Wirth M, Hebert J, Gold D (2018). Associations of prenatal and early life dietary inflammatory potential with childhood adiposity and cardiometabolic risk in Project Viva. Pediatr Obes.

[45] Winkvist A, Klingberg S, Nilsson LM, Wennberg M, Renström F, Hallmans G (2017). Longitudinal 10-year changes in dietary intake and associations with cardio-metabolic risk factors in the Northern Sweden Health and Disease Study. Nutr J.

[46] Tyrovolas S, Koyanagi A, Kotsakis GA, Panagiotakos D, Shivappa N, Wirth MD (2017). Dietary inflammatory potential is linked to cardiovascular disease risk burden in the US adult population. Int J Cardiol.

[47] Ruiz-Canela M, Zazpe I, Shivappa N, Hebert JR, Sanchez-Tainta A, Corella D (2015). Dietary inflammatory index and anthropometric measures of obesity in a population sample at high cardiovascular risk from the PREDIMED (PREvencion con DIeta MEDiterranea) trial. Br J Nutr.

[48] Camargo-Ramos CM, Correa-Bautista JE, Correa-Rodríguez M, Ramírez-Vélez R (2017). Dietary inflammatory index and cardiometabolic risk parameters in overweight and sedentary subjects. Int J Environ Res Public Health.

[49] Cantero I, Abete I, Babio N, Arós F, Corella D, Estruch R (2017). Dietary Inflammatory Index and liver status in subjects with different adiposity levels within the PREDIMED trial. Clin Nutr.

[50] Tabung FK, Smith-Warner SA, Chavarro JE, Fung TT, Hu FB, Willett WC (2017). An empirical dietary inflammatory pattern score enhances prediction of circulating inflammatory biomarkers in adults. J Nutr.

[51] Deng FE, Shivappa N, Tang Y, Mann JR, Hebert JR (2017). Association between diet-related inflammation, all-cause, all-cancer, and cardiovascular disease mortality, with special focus on prediabetics: findings from NHANES III. Eur J Nutr.

[52] Neufcourt L, Assmann KE, Fezeu LK, Touvier M, Graffouillère L, Shivappa N (2016). Prospective association between the dietary inflammatory index and cardiovascular diseases in the Supplémentation en Vitamines et Minéraux Antioxydants (SU. VI. MAX) Cohort. J Am Heart Assoc..

[53] O’Neil A, Shivappa N, Jacka FN, Kotowicz MA, Kibbey K, Hebert JR (2015). Pro-inflammatory dietary intake as a risk factor for CVD in men: a 5-year longitudinal study. Br J Nutr.

[54] Shivappa N, Blair CK, Prizment AE, Jacobs DR, Steck SE, Hébert JR (2016). Association between inflammatory potential of diet and mortality in the Iowa Women’s Health study. Eur J Nutr.

[55] Shivappa N, Harris H, Wolk A, Hebert JR (2016). Association between inflammatory potential of diet and mortality among women in the Swedish Mammography Cohort. Eur J Nutr.

[56] Shivappa N, Steck SE, Hussey JR, Ma Y, Hebert JR (2017). Inflammatory potential of diet and all-cause, cardiovascular, and cancer mortality in National Health and Nutrition Examination Survey III Study. Eur J Nutr.

[57] Shivappa N, Tavani A, Hébert JR, Rosato V, La Vecchia C (2017). Dietary inflammatory index and acute myocardial infarction in a large Italian case–control study. Eur J Public Health.

[58] Abdurahman AA, Azadbakhat L, Rasouli M, Chamari M, Qorbani M, Dorosty AR (2019). Association of dietary inflammatory index with metabolic profile in metabolically healthy and unhealthy obese people. Nutr Dietetics.

[59] Andrade PA, Hermsdorff HHM, Leite JIA, Shivappa N, Hébert JR, Henriques HKF (2019). Baseline pro-inflammatory diet is inversely associated with change in weight and body fat 6 months following-up to bariatric surgery. Obes Surg.

[60] Aslani Z, Qorbani M, Hébert JR, Shivappa N, Motlagh ME, Asayesh H (2019). Association of Dietary Inflammatory Index with anthropometric indices in children and adolescents: the weight disorder survey of the Childhood and Adolescence Surveillance and Prevention of Adult Non-communicable Disease (CASPIAN)-IV study. Br J Nutr.

[61] Carvalho CA, Silva AAM, Assunção MCF, Fonseca PCA, Barbieri MA, Bettiol H (2019). The dietary inflammatory index and insulin resistance or metabolic syndrome in young adults. Nutrition.

[62] Phillips C, Shivappa N, Hébert J, Perry I (2018). Dietary inflammatory index and biomarkers of lipoprotein metabolism, inflammation and glucose homeostasis in adults. Nutrients.

[63] Correa-Rodríguez M, Rueda-Medina B, González-Jiménez E, Correa-Bautista JE, Ramírez-Vélez R, Schmidt-RioValle J (2018). Dietary inflammatory index, bone health and body composition in a population of young adults: a cross-sectional study. Int J Food Sci Nutr.

[64] Denova-Gutiérrez E, Muñoz-Aguirre P, Shivappa N, Hébert J, Tolentino-Mayo L, Batis C (2018). Dietary inflammatory index and type 2 diabetes mellitus in adults: the diabetes mellitus survey of Mexico City. Nutrients.

[65] Farhangi MA, Najafi M (2018). Dietary inflammatory index: a potent association with cardiovascular risk factors among patients candidate for coronary artery bypass grafting (CABG) surgery. Nutr J.

[66] Muhammad HFL, van Baak MA, Mariman EC, Sulistyoningrum DC, Huriyati E, Lee YY (2019). Dietary inflammatory index score and its association with body weight, blood pressure, lipid profile, and leptin in indonesian adults. Nutrients.

[67] Alam I, Shivappa N, Hebert JR, Pawelec G, Larbi A (2018). Relationships between the inflammatory potential of the diet, aging and anthropometric measurements in a cross-sectional study in Pakistan. Nutr Healthy Aging.

[68] Kim H-Y, Lee J, Kim J (2018). Association between dietary inflammatory index and metabolic syndrome in the general korean population. Nutrients.

[69] Correa-Rodríguez M, González-Jiménez E, Rueda-Medina B, Tovar-Gálvez MI, Ramírez-Vélez R, Correa-Bautista JE (2018). Dietary inflammatory index and cardiovascular risk factors in Spanish children and adolescents. Res Nurs Health.

[70] Mazidi M, Shivappa N, Wirth MD, Hebert JR, Mikhailidis DP, Kengne AP (2018). Dietary inflammatory index and cardiometabolic risk in US adults. Atherosclerosis.

[71] Mirmajidi S, Izadi A, Saghafi-Asl M, Vahid F, Karamzad N, Amiri P (2019). Inflammatory potential of diet: association with chemerin, omentin, lipopolysaccharide-binding protein, and insulin resistance in the apparently healthy obese. J Am Coll Nutr.

[72] San KMM, Fahmida U, Wijaksono F, Lin H, Zaw KK, Htet MK (2018). Chronic low grade inflammation measured by dietary inflammatory index and its association with obesity among school teachers in Yangon. Myanmar Asia Pac J Clin Nutr.

[73] Nikniaz L, Nikniaz Z, Shivappa N, Hébert JR (2018). The association between dietary inflammatory index and metabolic syndrome components in Iranian adults. Prim Care Diabetes.

[74] Shivappa N, Hébert JR, Akhoundan M, Mirmiran P, Rashidkhani B (2019). Association between inflammatory potential of diet and odds of gestational diabetes mellitus among Iranian women. J Maternal-Fetal Neonatal Med.

[75] Park S, Na W, Sohn C (2018). Relationship between osteosarcopenic obesity and dietary inflammatory index in postmenopausal Korean women: 2009 to 2011 Korea National Health and Nutrition Examination Surveys. J Clin Biochem Nutr.

[76] Ren Z, Zhao A, Wang Y, Meng L, Szeto I, Li T (2018). Association between dietary inflammatory index, C-reactive protein and metabolic syndrome: a cross-sectional study. Nutrients.

[77] Hodge AM, Bassett JK, Dugué P-A, Shivappa N, Hébert JR, Milne R (2018). Dietary inflammatory index or Mediterranean diet score as risk factors for total and cardiovascular mortality. Nutr Metab Cardiovasc Dis.

[78] Park YMM, Choi MK, Lee SS, Shivappa N, Han K, Steck SE (2019). Dietary inflammatory potential and risk of mortality in metabolically healthy and unhealthy phenotypes among overweight and obese adults. Clin Nutr..

[79] Park S-Y, Kang M, Wilkens L, Shvetsov Y, Harmon B, Shivappa N (2018). The Dietary inflammatory index and all-cause, cardiovascular disease, and cancer mortality in the multiethnic cohort study. Nutrients.

[80] Coussens LM, Werb Z (2002). Inflammation and cancer. Nature.

[81] Festa A, D’Agostino R, Howard G, Mykkanen L, Tracy RP, Haffner SM (2000). Chronic subclinical inflammation as part of the insulin resistance syndrome the Insulin Resistance Atherosclerosis Study (IRAS). Circulation.

[82] Ridker PM, Cushman M, Stampfer MJ, Tracy RP, Hennekens CH (1997). Inflammation, aspirin, and the risk of cardiovascular disease in apparently healthy men. N Engl J Med.

[83] Shacter E, Weitzman SA (2002). Chronic inflammation and cancer. Oncology.

[84] Wang X, Bao W, Liu J (2013). (2013) Inflammatory markers and risk of type 2 diabetes a systematic review and meta-analysis. Diabetes Care.

