# Correction to: Association of dietary inflammatory potential with cardiometabolic risk factors and diseases: a systematic review and dose–response meta-analysis of observational studies

**DOI:** 10.1186/s13098-020-00615-2

**Published:** 2020-12-03

**Authors:** Zahra Aslani, Omid Sadeghi, Motahar Heidari-Beni, Hoda Zahedi, Fereshteh Baygi, Nitin Shivappa, James R. Hébert, Sajjad Moradi, Gity Sotoudeh, Hamid Asayesh, Shirin Djalalinia, Mostafa Qorbani

**Affiliations:** 1 Department of Community Nutrition, School of Nutritional Sciences and Dietetics, Tehran University of Medical Sciences, Tehran, Iran; 2 Students’ Scientific Research Center, Tehran University of Medical Sciences, Tehran, Iran; 3 Child Growth and Development Research Center, Research Institute for Primordial Prevention of Non-Communicable Disease, Isfahan University of Medical Sciences, Isfahan, Iran; 4 Hematopoietic Stem Cell Research Center, Shahid Beheshti University of Medical Sciences, Tehran, Iran; 5 Center of Maritime Health and Society, Department of Public Health, University of Southern Denmark, Esbjerg, Denmark; 6 Cancer Prevention and Control Program, University of South Carolina, Columbia, SC 29208 USA; 7 Department of Epidemiology and Biostatistics, Arnold School of Public Health, University of South Carolina, Columbia, SC 29208 USA; 8 Connecting Health Innovations LLC, Columbia, SC 29201 USA; 9 Halal Research Center of IRI, FDA, Tehran, Iran; 10 Department of Clinical Nutrition, School of Nutrition and Food Science, Isfahan University of Medical Sciences, Isfahan, Iran; 11 Department of Medical Emergencies, Qom University of Medical Sciences, Qom, Iran; 12 Development of Research & Technology Center, Ministry of Health and Medical Education, Tehran, Iran; 13 Non-Communicable Diseases Research Center, Endocrinology and Metabolism Population Sciences Institute, Tehran University of Medical Sciences, Tehran, Iran; 14 Non-Communicable Diseases Research Center, Alborz University of Medical Sciences, Karaj, Iran; 15 Chronic Diseases Research Center, Endocrinology and Metabolism Population Sciences Institute, Tehran University of Medical Sciences, Tehran, Iran

## Correction to: Diabetol Metab Syndr (2020) 12:86 https://doi.org/10.1186/s13098-020-00592-6

It was highlighted that in the original article, the Funding section statement was incorrect. This Correction article shows the incorrect and correct Funding section statement. The original article has been updated.

**Incorrect Funding statement**

This research did not receive any special grant funding.

**Correct Funding statement**

This study was supported by National Institute for Medical Research Development (NIMAD), Grant No. 971036.
