# Supplementary material for: Association of dietary inflammatory potential with cardiometabolic risk factors and diseases: a systematic review and dose–response meta-analysis of observational studies
Source: Diabetol Metab Syndr. 2020 Oct 7;12:86. doi: 10.1186/s13098-020-00592-6 (PMC7590706; doi:10.1186/s13098-020-00592-6)
Supplement: Supplementary file 2 — Additional file 2: Table S1. Search strategy in PubMed. [file 13098_2020_592_MOESM2_ESM.docx]

| Cardio-metabolic | Dietary inflammatory index | **Supplementary Table 1.** Search strategy in PubMed |
| --- | --- | --- |
| - | - | Mesh term |
| #10:Cardiometabolic [Title and Abstract]  #11 :Cardio-metabolic [Title and Abstract]  #12:"Cardiovascular disease*" [Title and Abstract]  #13:CVD [Title and Abstract]  #14:Diabete* [Title and Abstract]  #15:"Metabolic syndrome*"[Title and Abstract]  #16: "Metabolic X Syndrome"[Title and Abstract]  #17: "Dysmetabolic Syndrome"[Title and Abstract]  #18:Dyslipidemia [Title and Abstract]  #19:Hypertension [Title and Abstract]  #20:"Body mass index" [Title and Abstract]  #21: BMI [Title and Abstract]  #22: "Waist circumference" [Title and Abstract]  #23: WC [Title and Abstract]  #24: Lipid* [Title and Abstract]  #25: "Lipid profile"[Title and Abstract]  #26:"Lipid panel"[Title and Abstract]  #27: "Lipid_profile"[Title and Abstract]  #28: Hyperlipidemia[Title and Abstract]  #29: Lipoprotein[Title and Abstract]  #30: Cholesterol[Title and Abstract]  #31:TC[Title and Abstract]  #32:Triglyceride[Title and Abstract]  #33:TG[Title and Abstract]  #34: "Low density lipoprotein"[Title and Abstract]  #35:LDL[Title and Abstract]  #36:"High density lipoprotein"[Title and Abstract]  #37:HDL[Title and Abstract]  #38: "Blood pressure" [Title and Abstract]  #39: BP [Title and Abstract]  #40: Glucose [Title and Abstract]  #41:"Glucose Homeostasis"[Title and Abstract]  #42:"Fasting Blood Sugar"[Title and Abstract]  #43: FBS[Title and Abstract]  #44:"Fasting Plasma glucose"[Title and Abstract]  #45:FPG[Title and Abstract]  #46: Insulin [Title and Abstract]  #47: "Insulin Resistance Syndrome"[Title and Abstract]  #48: "Elevated blood sugar" [Title and Abstract]  #49: "Metabolic disease*" [Title and Abstract]  #50:"Chronic disease*"[Title and Abstract]  #51:Stroke [Title and Abstract]  #52:Obesity[Title and Abstract]  #53:"Abdominal obesity"[Title and Abstract]  #54: Overweight[Title and Abstract]  #55: #10 OR #11 OR #12 OR #13 OR #14 OR #15 OR #16 OR #17 OR #18 OR #19 OR #20 OR #21 OR #22 OR #23 OR #24 OR #25 OR #26 OR #27 OR #28 OR #29 OR #30 OR #31 OR #32 OR #33 OR #34 OR #35 OR #36 OR #37 OR #38 OR #39 OR #40 OR #41 OR #42 OR #43 OR #44 OR #45 OR #46 OR #47 OR #48 OR #49 OR #50 OR #51 OR #52 OR#53 OR #54  #56: #9 AND #55 | #1: "Dietary inflammatory index"[Title/Abstract]  #2: "Diet-related inflammation"[Title/Abstract]    #3: "Dietary inflammatory potential"[Title/Abstract]  #4: "Diet score*"[Title/Abstract]  #5: "Dietary inflammation" [Title/Abstract]  #6:"Index-based dietary patterns" [Title/Abstract]  #7:"Diet-borne systemic inflammation" [Title/Abstract]  #8:"Inflammatory diet" [Title/Abstract]  #9: #1 OR #2 OR #3 OR #4 OR #5 OR #6 OR #7 OR #8 | Text words |
