# Supplementary figures and images for: Association of dietary inflammatory potential with cardiometabolic risk factors and diseases: a systematic review and dose–response meta-analysis of observational studies
Source: Diabetol Metab Syndr. 2020 Oct 7;12:86. doi: 10.1186/s13098-020-00592-6 (PMC7590706; doi:10.1186/s13098-020-00592-6)

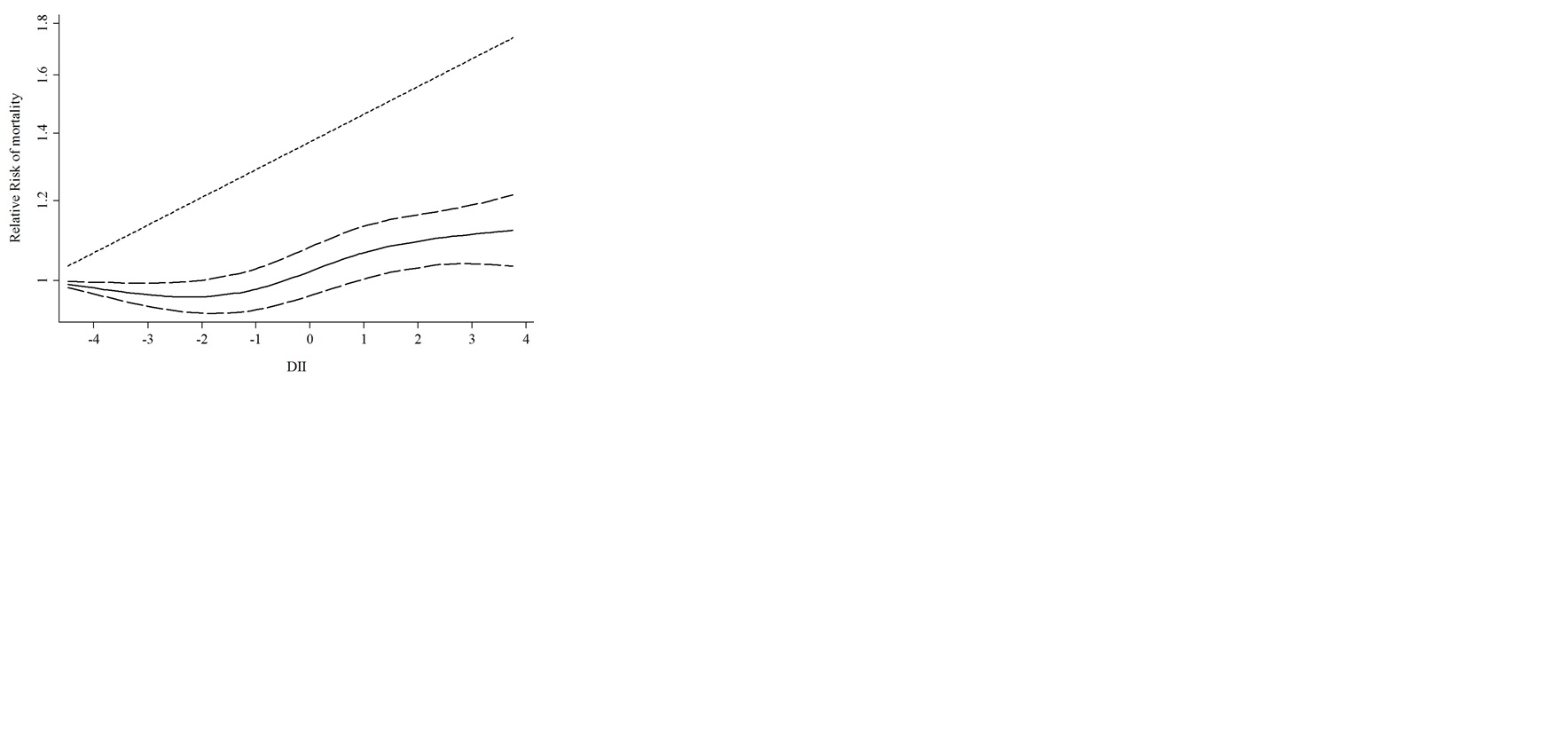

Supplement: Supplementary file 3 — Additional file 3: Figure S1. Dose–response association between the DII and risk of cardiometabolic diseases mortality. [file 13098_2020_592_MOESM3_ESM.jpg]

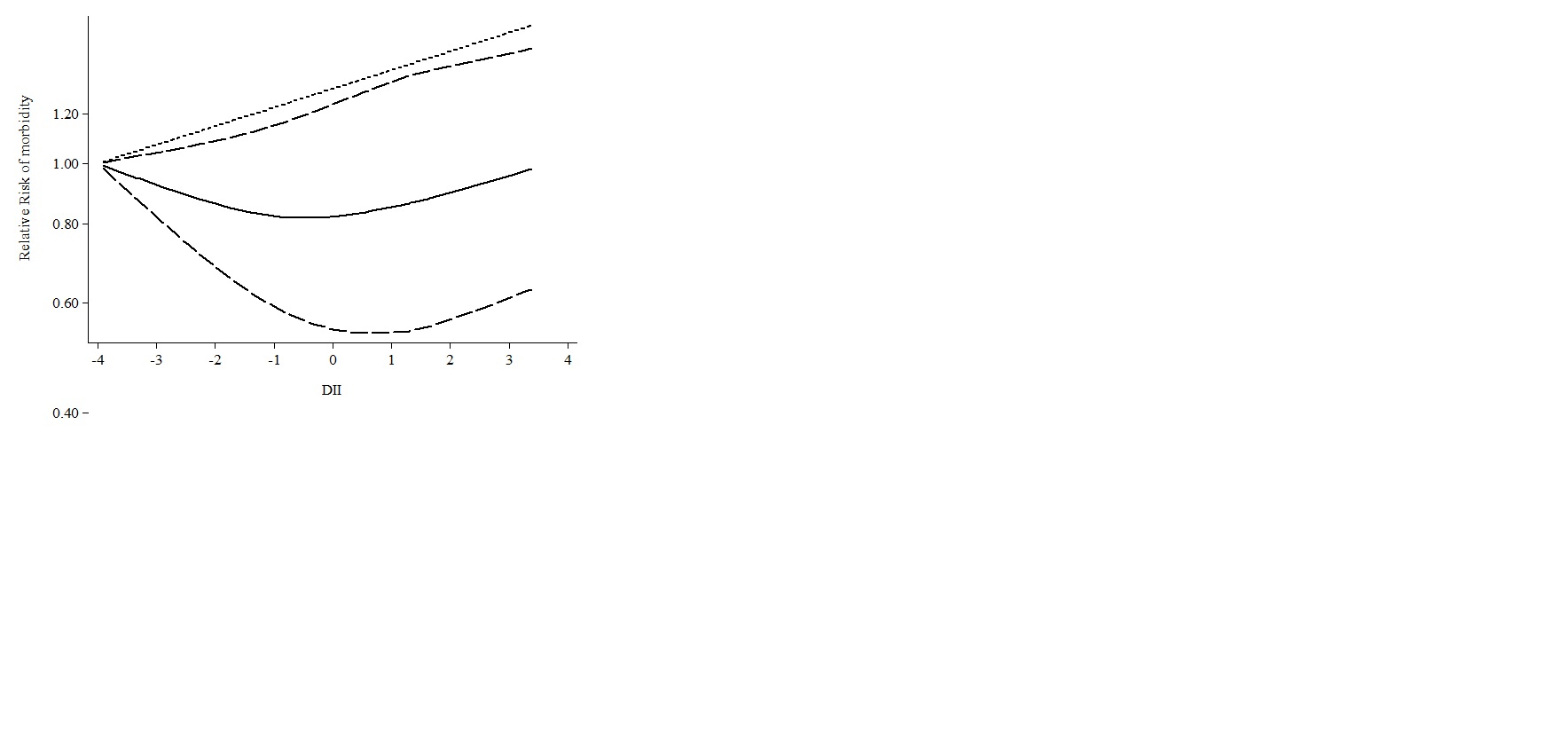

Supplement: Supplementary file 4 — Additional file 4: Figure S2. Dose–response association between the DII and risk of cardiometabolic diseases. [file 13098_2020_592_MOESM4_ESM.jpg]

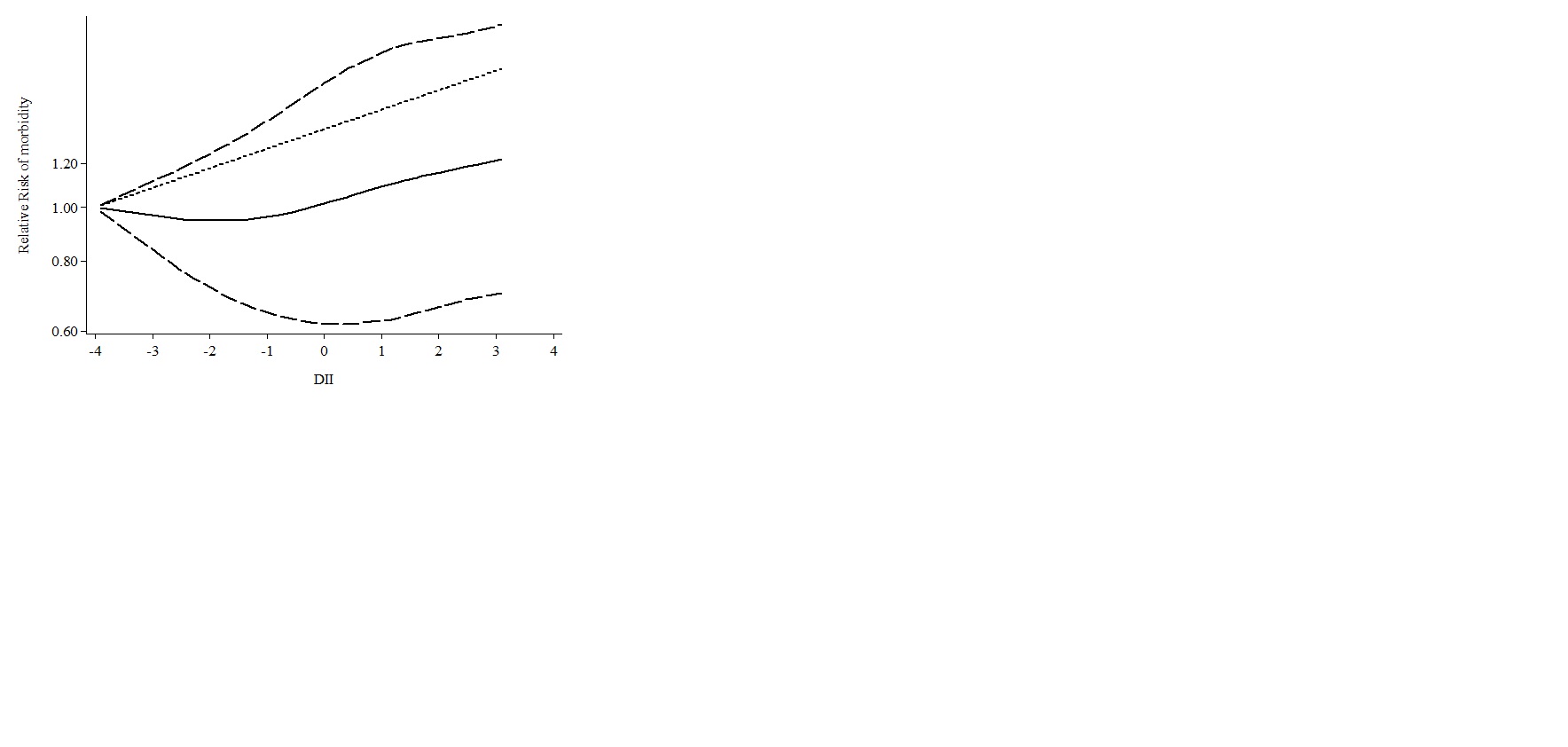

Supplement: Supplementary file 5 — Additional file 5: Figure S3. Dose–response association between the DII and risk of cardiometabolic diseases in cohort studies. [file 13098_2020_592_MOESM5_ESM.jpg]

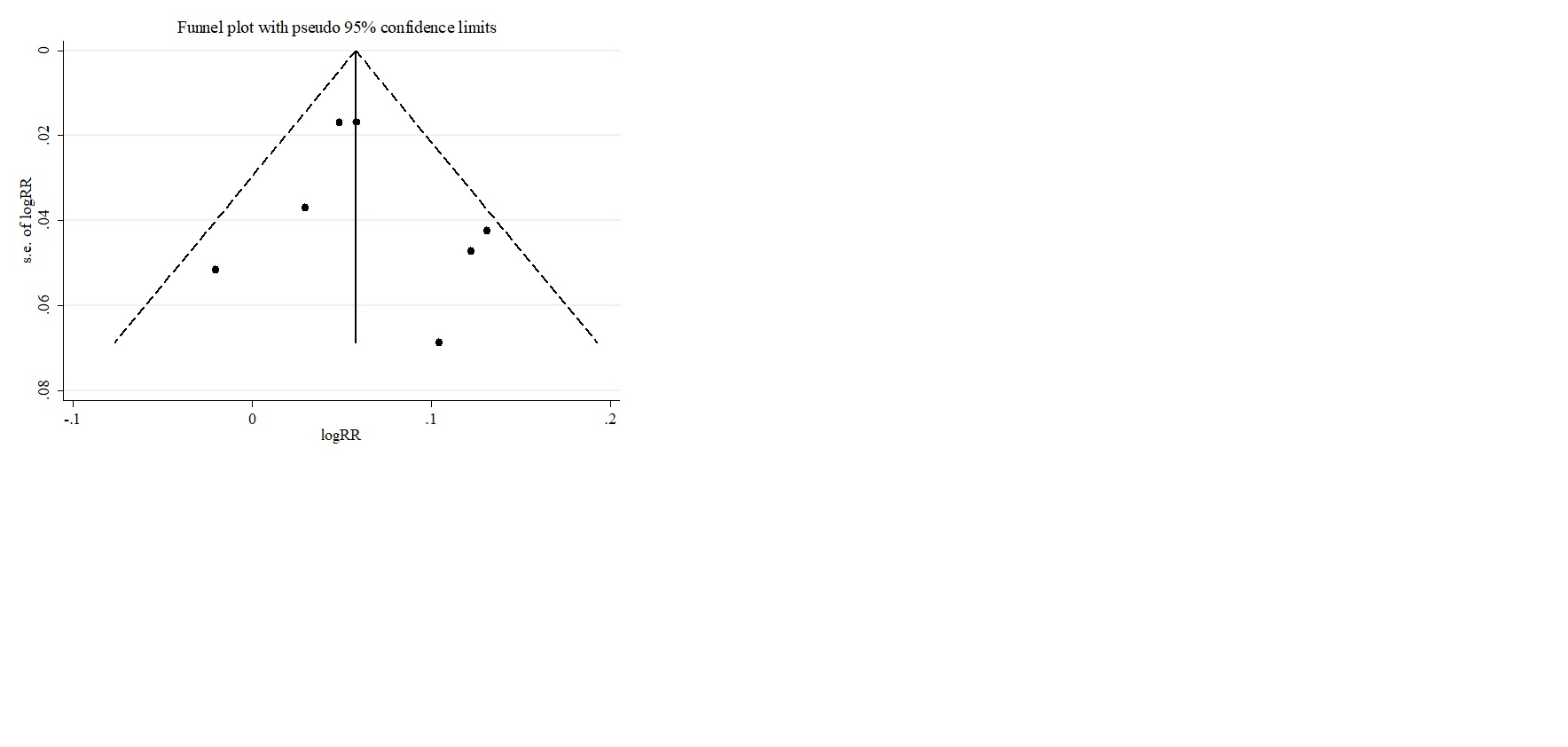

Supplement: Supplementary file 6 — Additional file 6: Figure S4. Funnel plot of dietary inflammatory index (DII) (as a continuous variable) with risk of cardiometabolic diseases. [file 13098_2020_592_MOESM6_ESM.jpg]

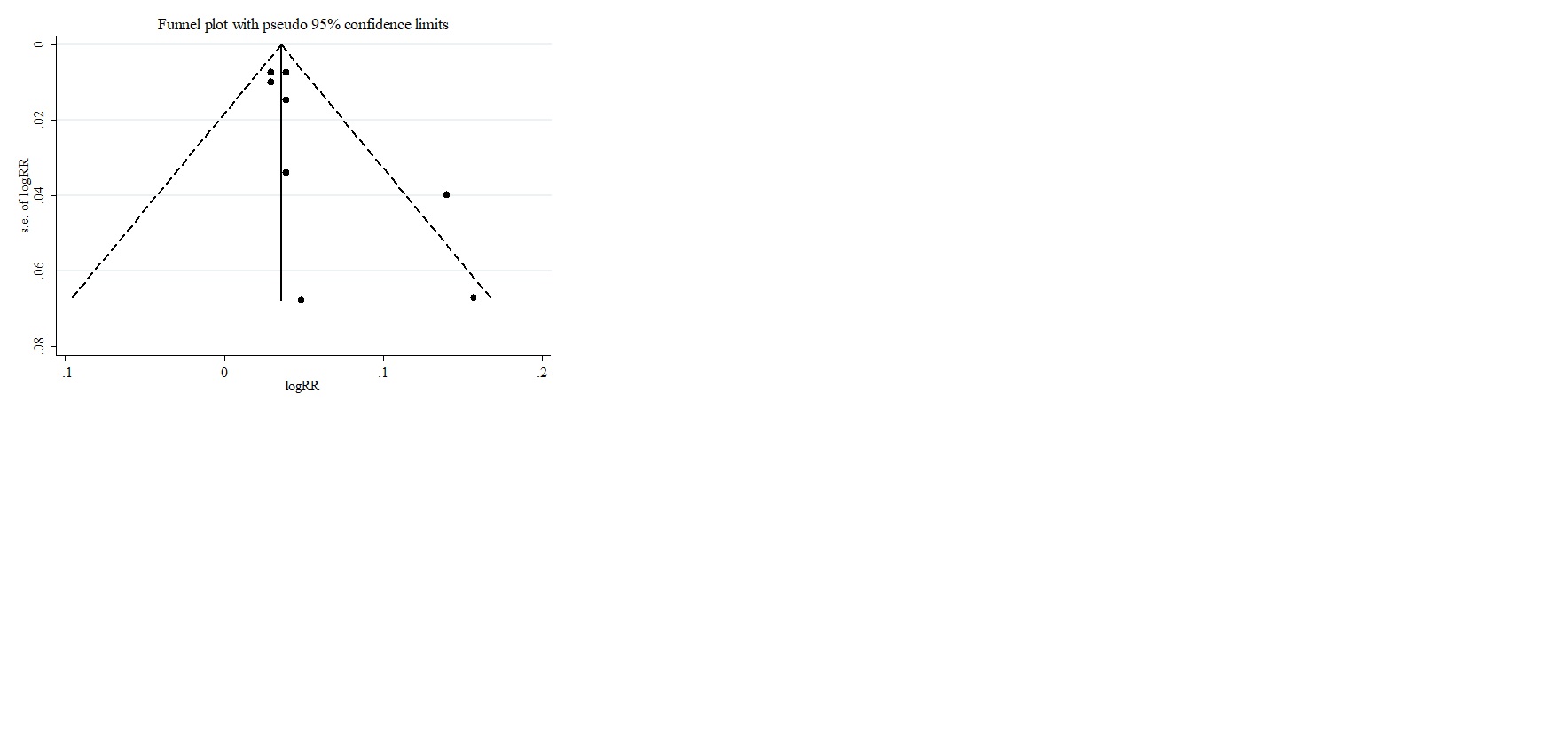

Supplement: Supplementary file 7 — Additional file 7: Figure S5. Funnel plot of dietary inflammatory index (DII) (as a continuous variable) with risk of cardiometabolic diseases mortality. [file 13098_2020_592_MOESM7_ESM.jpg]

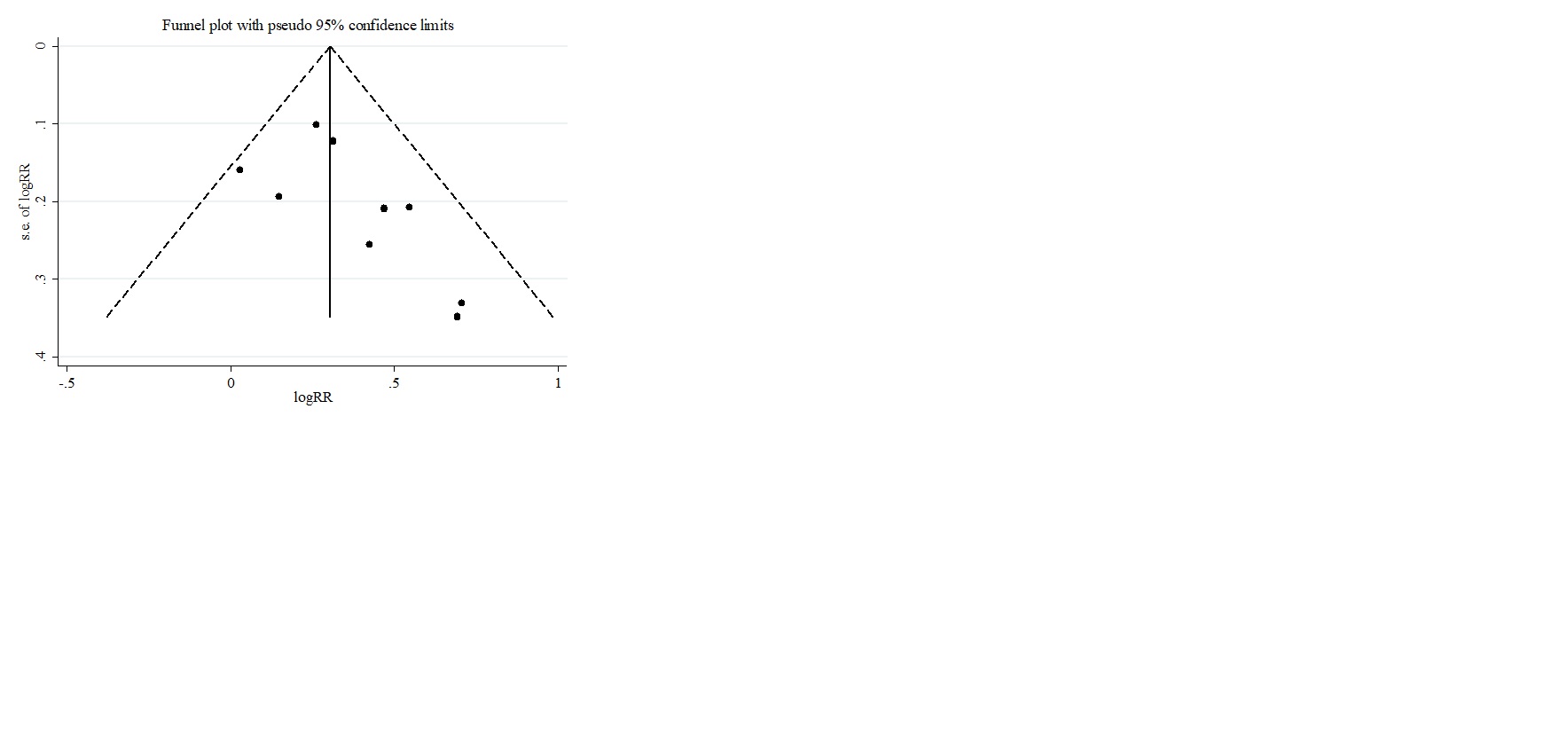

Supplement: Supplementary file 8 — Additional file 8: Figure S6. Funnel plot of dietary inflammatory index (DII) (as a categorical variable) with risk of cardiometabolic diseases. [file 13098_2020_592_MOESM8_ESM.jpg]

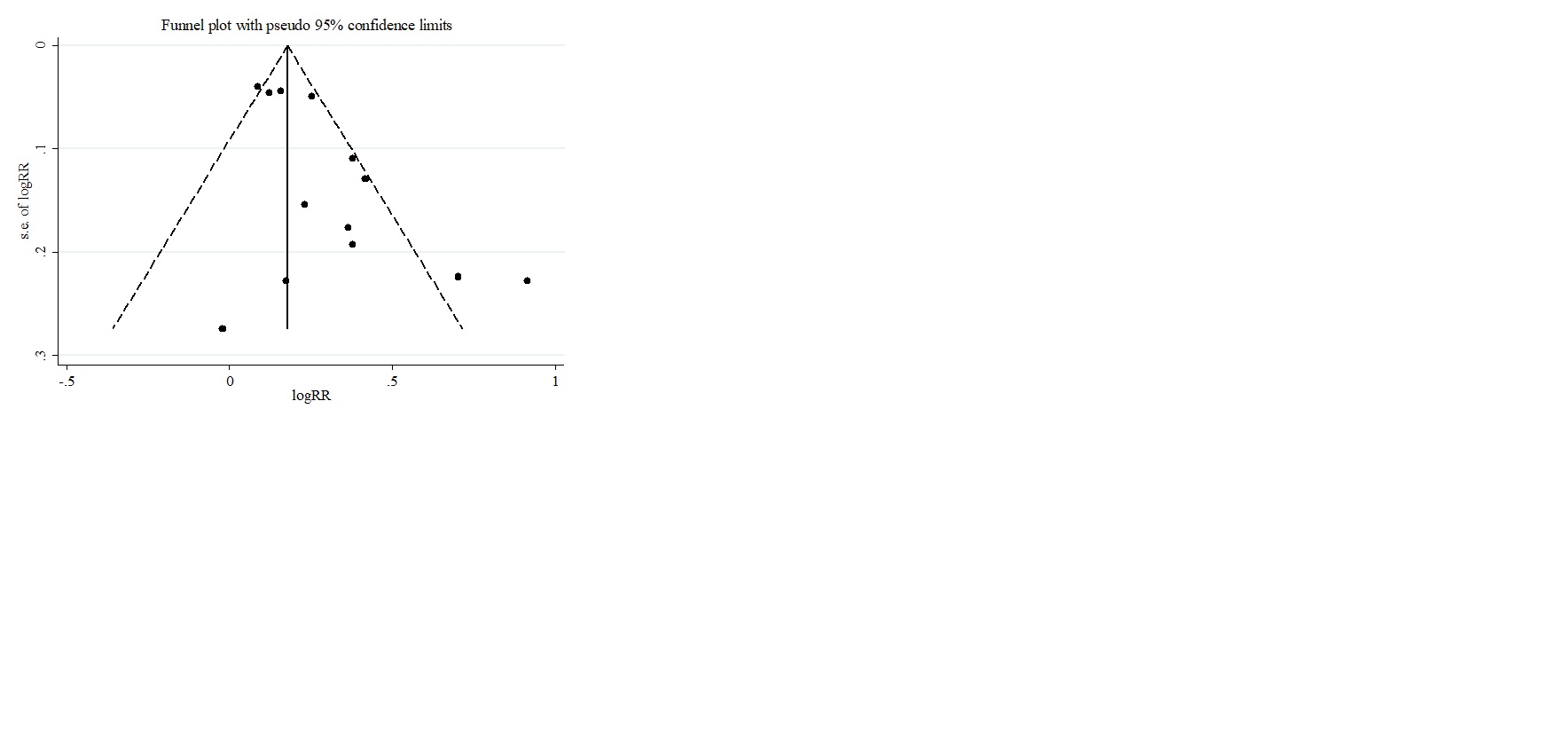

Supplement: Supplementary file 9 — Additional file 9: Figure S7. Funnel plot of dietary inflammatory index (DII) (as a categorical variable) with risk of cardiometabolic diseases mortality. [file 13098_2020_592_MOESM9_ESM.jpg]
